# Mucin-microbiome signatures shape the tumor microenvironment in gastric cancer

**DOI:** 10.1186/s40168-023-01534-w

**Published:** 2023-04-21

**Authors:** Baptiste Oosterlinck, Hannah Ceuleers, Wout Arras, Joris G. De Man, Karen Geboes, Heiko De Schepper, Marc Peeters, Sarah Lebeer, Jurgita Skieceviciene, Georgina L. Hold, Juozas Kupcinskas, Alexander Link, Benedicte Y. De Winter, Annemieke Smet

**Affiliations:** 1 Laboratory of Experimental Medicine and Paediatrics, Faculty of Medicine and Health Sciences, University of Antwerp, Universiteitsplein 1, 2610 Antwerp, Wilrijk Belgium; 2 Infla-Med Research Consortium of Excellence, University of Antwerp, Antwerp, Belgium; 3 Pathology Department, Gent University Hospital, Ghent, Belgium; 4 Division of Gastroenterology and Hepatology, Antwerp University Hospital, Edegem, Belgium; 5 Department of Oncology, Antwerp University Hospital, Edegem, Belgium; 6 Department of Bioscience Engineering, University of Antwerp, Antwerp, Belgium; 7 Department of Gastroenterology and Institute for Digestive Research, Lithuanian University of Health Sciences, Kaunas, Lithuania; 8 Microbiome Research Centre, St George and Sutherland Clinical School, University of New South Wales, Sydney, NSW Australia; 9 Department of Gastroenterology, Hepatology and Infectious Diseases, Otto-Von-Guericke University, Magdeburg, Germany

**Keywords:** Stomach cancer, Mucin, Microbiota, Survival

## Abstract

**Background and aims:**

We aimed to identify mucin-microbiome signatures shaping the tumor microenvironment in gastric adenocarcinomas and clinical outcomes.

**Methods:**

We performed high-throughput profiling of the mucin phenotypes present in 108 gastric adenocarcinomas and 20 functional dyspepsia cases using validated mucin-based RT-qPCRs with subsequent immunohistochemistry validation and correlated the data with clinical outcome parameters. The gastric microbiota was assessed by 16S rRNA gene sequencing, taxonomy, and community composition determined, microbial networks analyzed, and the metagenome inferred in association with mucin phenotypes and expression.

**Results:**

Gastric adenocarcinomas with an intestinal mucin environment or high-level *MUC13* expression are associated with poor survival. On the contrary, gastric MUC5AC or MUC6 abundance was associated with a more favorable outcome. The oral taxa *Neisseria, Prevotella*, and* Veillonella* had centralities in tumors with intestinal and mixed phenotypes and were associated with MUC13 overexpression, highlighting their role as potential drivers in MUC13 signaling in GC. Furthermore, dense bacterial networks were observed in intestinal and mixed mucin phenotype tumors whereas the lowest community complexity was shown in null mucin phenotype tumors due to higher *Helicobacter* abundance resulting in a more decreased diversity. Enrichment of oral or intestinal microbes was mucin phenotype dependent. More specifically, intestinal mucin phenotype tumors favored the establishment of pro-inflammatory oral taxa forming strong co-occurrence networks.

**Conclusions:**

Our results emphasize key roles for mucins in gastric cancer prognosis and shaping microbial networks in the tumor microenvironment. Specifically, the enriched oral taxa associated with aberrant MUC13 expression can be potential biomarkers in predicting disease outcomes.

Video Abstract

**Supplementary Information:**

The online version contains supplementary material available at 10.1186/s40168-023-01534-w.

## Background

Gastric cancer (GC) is the fifth most common cancer type and the fourth leading cause of cancer-related deaths worldwide [1]. The prognosis of GC remains poor due to the lack of symptoms in early disease, leading to a delayed diagnosis [2]. It is now widely accepted that GC is a multifactorial disease involving host genetic susceptibility and environmental factors, but the most frequent cause is infection by *Helicobacter pylori* (*H. pylori*) [3]. This class 1 human carcinogen plays a major role in the initial steps of the carcinogenic process through a hit-and-run mechanism priming the gastric mucosa for further oncogenic changes which are triggered by other microbial species [3, 4]. Studies assessing human gastric microbiota profiles have shown that dysbiosis in the stomach is a dynamic process correlating with cancer progression and that gastric adenocarcinomas are characterized by a decrease in *Helicobacter* abundance and enrichment of bacterial genera representing intestinal commensals (*Citrobacter*, *Clostridium*, *Lactobacillus*, *Achromobacter*, and *Rhodococcus*) or the oral microbiome (*Peptostreptococcus stomatis*, *Streptococcus anginosus*, *Parvimonas micra*, *Slackia exigua*, *Lactococcus*, and *Fusobacterium)* [5–8]. Furthermore, tumor microhabitats are not always as uniform as previously thought [8]. Besides changes in pH, mucin (MUC) expression, and distribution vary considerably among gastric tumor tissues with both gastric and intestinal mucins being widely expressed. Depending on the presence/absence of mucins, adenocarcinomas have been classified as having a gastric (i.e., tumors expressing only gastric MUC1, MUC5AC, and/or MUC6 mucins), intestinal (i.e., tumors expressing only intestinal MUC2, MUC3, MUC4, and/or MUC13 mucins), mixed (i.e., tumors expressing both gastric and intestinal mucins), or unclassified/null (i.e., tumors expressing nor gastric, nor intestinal mucins) mucin phenotype [9–15]. Early gastric cancers mainly exhibit a gastric mucin phenotype, whereas advanced cancers more frequently have an intestinal mucin phenotype. However, the clinical importance of mucin expression in gastric tumors is still controversial in the context of clinicopathological factors, such as disease outcome, as it remains unclear which mucin phenotype associates with a better or worse prognosis [9, 10, 12, 15–19]. Mucins are the gatekeepers of the mucus barrier covering the gastric epithelium and are expressed either as secretory or transmembrane glycoproteins [20–24]. Besides having a barrier function, they also serve as specialized niches for bacteria by acting as binding sites or metabolic substrates and are important determinants of site-specific bacterial colonization [20–24]. It has been suggested that aberrant mucin alterations due to neoplastic changes can result in the establishment of a new microbiota promoting tumor progression [7]. The differences in the abundance of arising new taxa (i.e., intestinal or oral) as previously described in GC [5, 25] may thus be assigned to the mucin phenotype (gastric, intestinal, mixed, or null mucin phenotype) of the tumor, but further investigation is required. To investigate the above hypothesis, we performed high-throughput profiling of the mucin phenotypes and bacterial communities present in 108 gastric adenocarcinoma and 20 functional dyspepsia (FD) (i.e., for comparison) cases using validated mucin-based RT-qPCRs with subsequent immunohistochemistry (IHC) validation and 16S rRNA gene sequencing integrated with clinical data to identify mucin-microbiota signatures associated with GC and clinical outcome.

## Methods

### Patients

A total of 108 GC patients undergoing gastrectomy for gastric cancer were enrolled in this study. Seventeen patients were recruited via the biobank of the Antwerp University Hospital (UZA; Belgium), 48 via the Department of Digestive Oncology of Ghent University Hospital (Belgium), and 43 via the Institute for Digestive Research of the Lithuanian University of Health Sciences (Lithuania). Twenty patients with FD undergoing gastroscopy for clinical reasons and showing no macroscopic abnormalities were included as a comparison cohort via the Department of Gastroenterology and Hepatology (UZA; Belgium). Tumor (*n* = 108) and adjacent non-tumor (*n* = 108) tissues from GC patients and biopsy tissues from FD patients were stored in RNAlater or snap frozen at − 80 °C or embedded in paraffin for RNA/DNA extraction with subsequent downstream approaches or IHC analyses, respectively. The recorded data for the GC cohorts included: gender, age, tumor localization, Lauren’s classification (intestinal, diffuse, or mixed histological subgroups) [26], TNM-G staging, and survival rate (Table S1). This study was approved by the Ethical Committee of the Antwerp University Hospital (EC 19/15/205 (GC) and B300201733550 (FD)) and the Kaunas Regional Ethics Committee (Protocol No—BE-2–10), and written informed consent was obtained from the patients prior to sample collection. Samples were registered and stored until analysis in the Biobank Antwerpen, Antwerp, Belgium (BE 71,030,031,000; BBMR-ERIC, Belgian no. access: 1, Last: April 10, 2021 [BIORESOURCE]).

### Mucin mRNA expression by RT-qPCR

The total RNA was extracted from tumor and adjacent non-tumor tissues and FD biopsies using the NucleoSpin RNA plus kit (Macherey–Nagel) following the manufacturer’s instructions. RNA concentration was evaluated using the nanodrop ND-1000 UV–Vis spectrophotometer (Thermo Fisher Scientific). Two hundred fifty nanograms of RNA was converted to cDNA by reverse transcription using the SensiFast cDNA synthesis kit (Bioline). Relative mucin gene expression was determined by SYBR Green RT-qPCR using validated QuantiTect primers (Qiagen, Table S2) and GoTaq qPCR master mix (Promega) using a QuantStudio 3 real-time PCR instrument (Thermo Fisher Scientific) [27]. Relative mRNA expression of mucin genes was normalized to the expression of *ACTB* and *GAPDH* housekeeping genes using qbase + software (Biogazelle), which implements an adapted ΔCt method and calculates calibrated normalized relative quantities (CNRQ) for downstream analysis [28]. To define the mucin phenotypes in the tumor tissues and to clarify conflicting results previously obtained with IHC regarding the clinical importance of mucin expression, we designed a new approach based on RT-qPCR [9, 10, 12, 15–19]. This quantitative technique displays a broader dynamic range with higher sensitivity and reproducibility and is significantly more specific than IHC resulting in more reliable data [29]. More specifically, the 90% confidence interval (CI) of the relative mRNA expression of each mucin was determined for the FD cohort. The lower (LL) and upper (UL) limits of CIs were used to stratify the relative expression levels found in the tumor tissue into three distinct classes: (1) low < LL, (2) LL < mid < UL, and (3) UL < high (Table S3). Depending on the class of relative mRNA expression of mucins, adenocarcinomas were classified as having a gastric (mid or high mRNA levels of MUC5AC, MUC6, and/or MUC1 and low mRNA levels of MUC2, MUC4, and MUC13), intestinal (low mRNA levels of gastric mucins and high mRNA levels of at least one intestinal mucin), mixed (mid or high mRNA levels of at least one gastric mucin and high mRNA levels of at least one intestinal mucin), or null (low mRNA levels for all gastric and intestinal mucins) mucin phenotype.

### Immunohistochemistry

To evaluate mucin mRNA expression at the protein level, tissue segments were fixed for 24 h in 4% formaldehyde and subsequently embedded in paraffin. Five-micrometer cross-sections were deparaffinized, rehydrated, and used for immunohistochemical staining using target-specific primary antibodies and visualization with a secondary streptavidin–horseradish peroxidase antibody and 3-amino-9-ethylcarbazole (AEC) substrate to detect the expression and localization of MUC1 (AF6298, R&D systems, 1:500), MUC2 (NBP1-31231, Novus Biologicals, 1:3000), MUC4 (NBP1-52193, Novus Biologicals, 1:3000), MUC5AC (ab3649, Abcam, 1:5000), MUC6 (ab216017, Abcam, 1:50), and MUC13 (MABC209, Merck Millipore, 1:1000). The stained sections were analyzed by light microscopy (Olympus BX43) [30]. Distinct staining in more than 10% of the gastric cells was recorded as positive immunoreactivity for the relevant mucin. Tumors containing epithelial cells expressing only gastric or intestinal-type mucins were classified as having a gastric or intestinal mucin phenotype, respectively. Those containing cells expressing both gastric and intestinal type mucins were classified as having a mixed phenotype, whereas tumors with cells expressing neither gastric nor intestinal type mucins were classified as having a null mucin phenotype. The degree of immunostaining was evaluated by two independent observers.

### Survival analysis

Kaplan–Meier curves and Cox proportional-hazards models were used for survival analysis. The Kaplan–Meier curves implementing the log-rank test were performed according to the tumor mucin phenotype and stratified mucin mRNA expression levels. The mucin phenotype and the six stratified mucin expression levels were also included in a Cox proportional-hazards model taking gender, age, tumor location, Lauren’s classification, and tumor stage into account.

### 16S rRNA gene sequencing

DNA was extracted from the gastric surgical specimens and biopsies using the DNeasy Blood and Tissue kit (Qiagen) following the manufacturer’s instructions. A negative and positive (ZymoBIOMICS Microbial Community DNA standard – cat no. D6305) control was also included and processed together with the samples. Subsequently, library preparation was performed according to the standard Illumina protocol for the V3 chemistry for paired-end sequencing (2 × 300 bp) using the universal forward 27Fmod, 5′AGRGTTHGATYMTGGCTCAG and reverse 338R, 5′TGCTGCCTCCCGTAGGAGT primers targeting the V1–V2 hypervariable region of the 16S rRNA gene [31]. All samples were pooled equimolarly and sequenced using a MiSeq Illumina platform.

### Sequence filtering and annotation

Sequence quality assessment, paired-end read merging, filtering, sequence denoising, and chimeric read filtering were performed using the dada2 R-package [32]. The final sequences were aligned to the SILVA reference database (version 138) and annotated using the DECIPHER R-package [33, 34]. Any sequences that were annotated as non-bacterial were discarded. Finally, sample rarefaction curves were determined and all samples with curves not attaining a stable plateau were considered to be undersampled and thus discarded. As a final filtering step, the counts from the negative control were subtracted from all samples after which the relative abundance was calculated. All genera with a relative abundance below 0.5% were pruned, and for the remaining genera, the original counts were used to recalculate the relative abundances [35]. Sample metadata, abundance table, taxonomy, phylogenetic tree, and reference sequence data were grouped into a Phyloseq object for downstream processing [36]. The alpha (chao1, observed richness, inverse Simpson, and Shannon) and beta diversity measures (Bray–Curtis and weighted unifrac) were calculated using the relative abundances. Differences in community composition were tested using analysis of similarities (ANOSIM) [37].

### Differential abundance analysis

Differential abundance analyses were done using the ALDEx2 R-package [38]. This package was selected based on its performance and native support for compositional datasets based on a recent review from Nearing et al. (2022) assessing different methods for differential abundance analysis [39]. All reference sequences of the amplicon sequence variants (ASV) found were compared against sequences from the Human oral Microbiome (HOM) database (HOMD 16S rRNA RefSeq version 15.22) and the Human Intestinal 16 s rRNA gene reference database using BLAST local nucleotide alignment [40, 41]. Only matches with at least 99% identity and over 250 base pairs were retained. ASVs with a species assigned and which were not annotated as having an oral or nasal habitat in the HOM database were retained as intestinal bacterial taxa. Relative abundances of both oral and intestinal species were compared between tumor, non-tumor, and FD tissues and among tumor samples with different mucin phenotypes to determine whether oral or intestinal taxa were enriched or depleted.

### Microbial network analysis

For calculating the co-occurrence and co-excluding microbial interactions, the co-occur R-package was used after the transformation of the count table to a presence/absence matrix [42]. The output generated is an object containing non-random associations with the probability of a lower and a higher co-occurrence for a genus pair. These probabilities were interpreted as *p* values for a negative or positive correlation, respectively [42]. Obtained networks were plotted using the ggraph R-package.

### Functional metagenome inference

The mucosa-associated functional metagenome was predicted using the PICRUSt2 algorithm with default settings [43]. PICRUSt2 is able to predict the presence of functional genes based on a set of reference genomes and a marker gene dataset, allowing for the prediction of pathway abundances among the GC cohorts. By default, the abundance of the enzyme commission (EC) number (= a numerical classification scheme for enzymes based on the chemical reactions they catalyze) is inferred based on the relative abundance of the genera. Subsequently, the EC numbers are transformed into MetaCyc pathways and allow for their abundance calculation. These MetaCyc pathway abundances were then used for a differential abundance analysis.

### Data analysis

Two sample and multiple group comparisons were done using the Wilcoxon rank sum test and the Kruskal–Wallis test, respectively. Spearman’s correlations between the clinical patient data, mucin mRNA expression levels, and bacterial abundancy in gastric adenocarcinoma samples were calculated. A correlogram plotting the Spearman’s rank correlation coefficient (*r*) between all parameter pairs was created. Differences in proportions were analyzed by Pearson’s *χ*^2^. After differential abundance analysis with ALDEx2, a Wilcoxon rank-sum test was used to assess significant differences between conditions. *P* values below 0.05 were considered significant. All analyses were done using R version 4.2.2 in RStudio.

## Results

### Phenotypical classification of gastric adenocarcinomas based on mucin expression

**Fig. 1:**
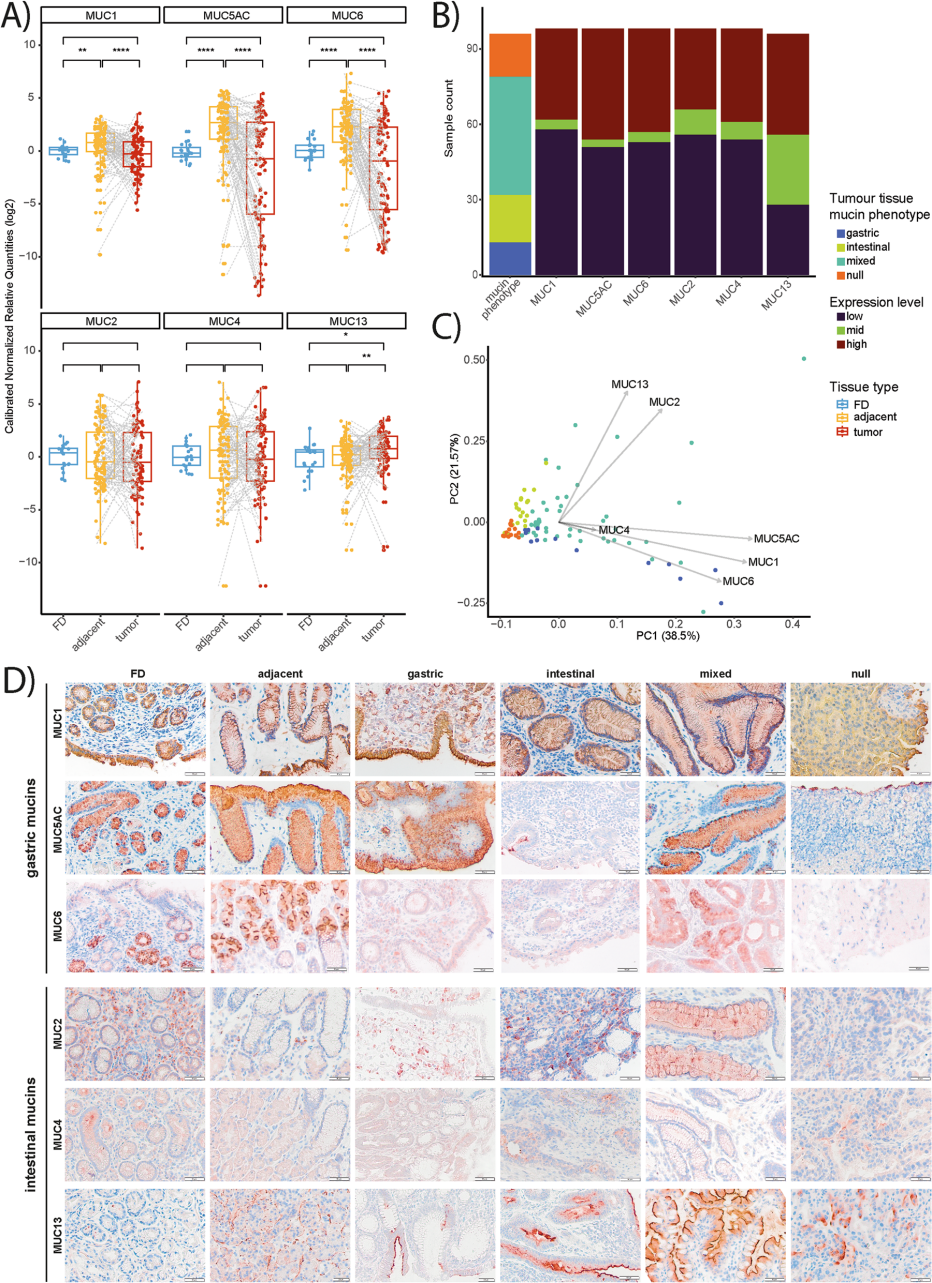
Aberrant mucin signatures in the stomach of GC patients compared to FD patients. **A** Relative mRNA expression of gastric (MUC1, MUC5AC, and MUC6; *n* = 100) and intestinal mucins (MUC2, MUC4, and MUC13; *n* = 100, 99, and 97, respectively) in gastric biopsies from FD patients (*n* = 20) and paired (highlighted by gray dashed lines) tumor and adjacent non-tumor tissues of 3 GC patient cohorts. Significant differences between FD, tumor, and adjacent non-tumor tissues are indicated by *P < 0.05, **P < 0.01, ***P < 0.001, and ****P < 0.0001 (Wilcoxon rank-sum test). **B** The bar sizes represent the number of patients per mucine phenotype (null, *n* = 13; intestinal, *n* = 19; mixed, *n* = 47; null, *n* = 17) or expression level (i.e., high, mid, or low expression of MUC1, MUC5AC, MUC6, MUC2, MUC4, and MUC13). For each mucin expression level, the mean CNRQ (SD) is shown here: MUC1, high: 3.01 (2.05), mid: 1.07 (0.08), low: 0.46 (0.29); MUC5AC, high: 10.8 (9.04), mid: 1.38 (0.03), low: 0.13 (0.22); MUC6, high: 9.72 (10.82), mid: 1.39 (0.2), low: 0.11 (0.2); MUC2, high: 15.94 (25.98), mid:1.35 (0.13), low: 0.33 (0.28); MUC4, high: 14.72 (22.91), mid: 1.29 (0.19), low: 0.35 (0.3); and MUC13, high: 5.27 (2.88), mid: 1.56 (0.33), low: 0.47 (0.3). **C** PCA plot based on mucin mRNA expression in tumor and adjacent non-tumor tissues of 3 GC patient cohorts (*n* = 97–100). PC1 explains 38.5% of the variation; PC2 explains 21.57% of the variation. **D** Immunohistochemistry was assessed to analyze MUC1, MUC2, MUC5AC, MUC4, MUC6, and MUC13 protein expression in the different tissue types (*n* = 5 per tissue type; i.e., FD, adjacent non-tumor, and gastric adenocarcinomas with gastric, intestinal, mixed, or null mucin phenotypes). Representative images were selected. Pictures were taken at 20 × magnification and scale bars are 20 µm or 50 µm

In the present study, we first analyzed the tumor and adjacent non-tumor tissues of the GC patient cohorts and the biopsy tissues of the FD patients to measure the relative mRNA expression of gastric (*MUC1, MUC5AC, MUC6*) and intestinal (*MUC2, MUC4, MUC13*) mucins. Overall, mRNA expression of *MUC1*, *MUC5AC*, and *MUC6* was significantly higher in the paired adjacent non-tumor tissues compared to the tumor and FD tissues (Fig. 1A). Regarding the intestinal mucins, a significant increase in *MUC13* mRNA expression was seen in the paired tumor tissues compared to the adjacent non-tumor and FD tissues whereas no significant alterations in expression were seen for *MUC2* and *MUC4* mRNA among the different sample types (Fig. 1A). The variable expression patterns of *MUC2* (i.e., marker for intestinal metaplasia) and *MUC4* (i.e., marker for spasmolytic polypeptide expressing metaplasia (SPEM)) seen in the adjacent non-tumor tissues suggest the presence of metaplastic changes in these peritumoral sites [44, 45]. The gastric adenocarcinomas were then subdivided in gastric, intestinal, mixed, or null mucin phenotype groups based on their relative mucin mRNA expression levels. Of the 108 tumor samples, 13 (12%) were classified in gastric, 19 (17,6%) in intestinal, 17 (15,7%) in null, and 47 (43,5%) in mixed mucin phenotype groups (Fig. 1B). From 12 samples (11.1%), the mucin phenotype could not be determined due to insufficient RNA quality (Fig. 1B). Subsequently, to validate the classification of the tumors in the different phenotype groups, a principal component analysis based on the mucin mRNA expression data was undertaken. Strikingly, *MUC2* and *MUC13* mRNA expression were the major determinants for tumors with an intestinal mucin phenotype whereas the expression of *MUC1*, *MUC5AC*, and *MUC6* mRNA were the best factors to identify tumors with a gastric mucin phenotype (Fig. 1C). Finally, mucin expression was also evaluated at the protein level by IHC further confirming our mRNA expression data (Fig. 1D).

### The intestinal mucin phenotype and aberrant MUC13 mRNA expression correlate with worse survival

We then evaluated collinearity between the mucin mRNA expression data, age, gender, tumor stage, Lauren’s classification, and survival using Spearman’s correlation tests (Fig. S1). A strong positive correlation was seen between *MUC13* mRNA expression and the Lauren’s intestinal phenotype whereas negative associations were noted between *MUC1* mRNA expression and survival and *MUC4* mRNA expression and age (Fig. S1). Furthermore, significant relationships among the gastric mucin mRNA expression profiles and the expression levels of the intestinal *MUC2* and *MUC4* were also identified (Fig. S1).

**Fig. 2:**
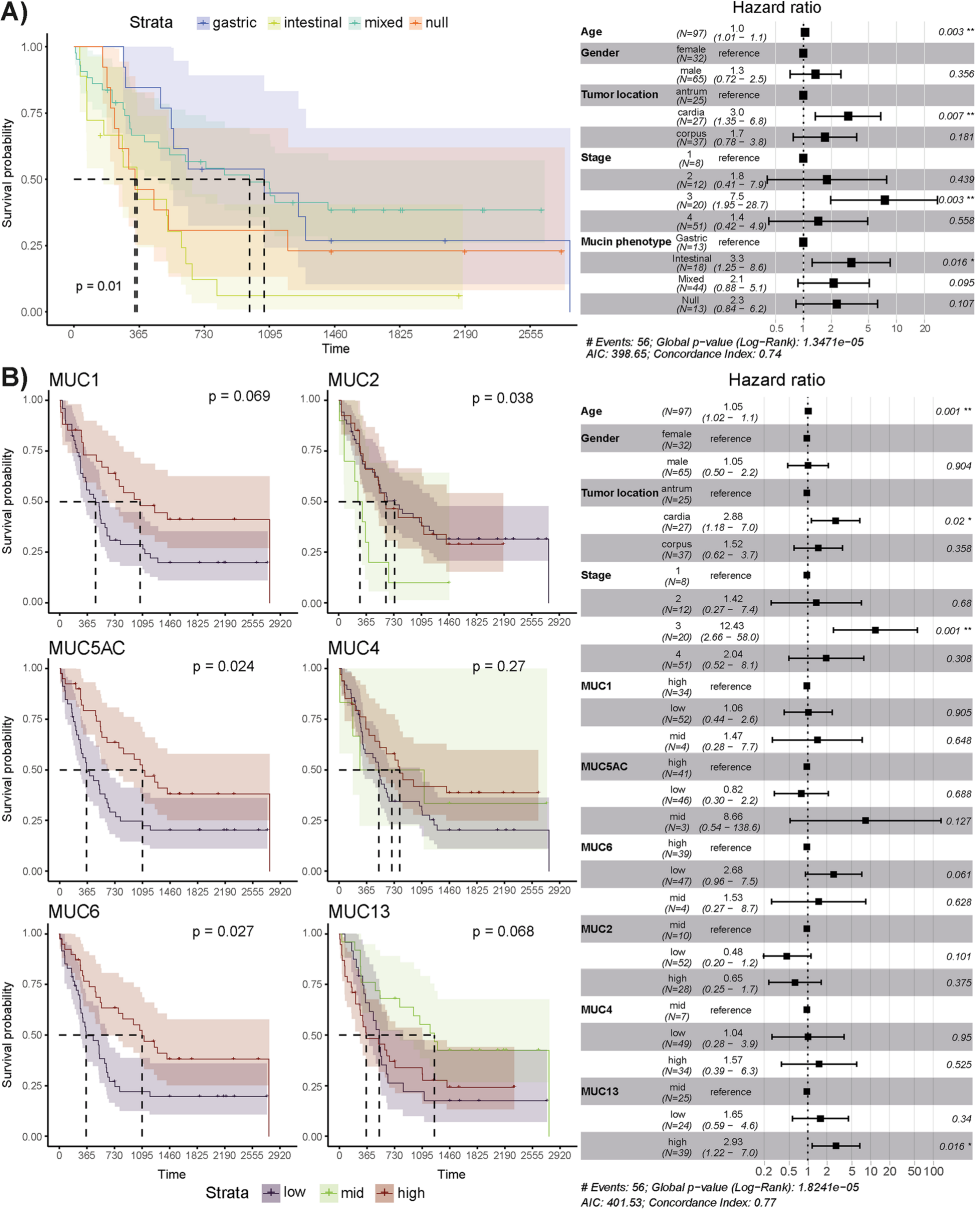
Intestinal mucin phenotype and aberrant MUC13 expression correlate with worse survival in GC patients.** A** Kaplan–Meier curve (left-hand side) and Cox-proportional hazards model (right-hand side) for survival analysis between patients with gastric tumors assigned to different mucin phenotypes (**A**) and showing low, mid, or high mRNA levels of a gastric (MUC1, MUC5AC, MUC6) or intestinal (MUC2, MUC4, MUC13) mucin (**B**). For the gastric mucins (MUC5AC, MUC6, and MUC1), the mid-level expression data was excluded due to a small number of observations (*n* ≤ 4). A forest plot of the Cox-proportional hazards model is shown (right). For the Kaplan–Meier curves (left), the *P* values were calculated using the log-rank test whereas the Wald test statistic was performed for the Cox-proportional hazards model (*n* for each group is shown on the forest plot)

Subsequently, associations between mucin mRNA expression, mucin phenotypes, and the 5-year survival rate were also investigated using Kaplan–Meier and Cox proportional hazards models (Fig. 2). For the latter model, clinical patient data (age, gender, tumor stage, and location; Table S1) was also taken into account. Both methods showed an association between the intestinal mucin phenotype and a worse survival rate compared to the gastric, mixed, and null mucin phenotypes (log-rank test, *P* = 0.01; Wald test, *P* = 0.016, Fig. 2A). Significant associations between individual mucin expression levels and survival rate were also identified. Specifically, Kaplan–Meier curves indicated that low mRNA expression of *MUC5AC* and *MUC6* in gastric tumor tissue correlated with worse survival (*P* ≤ 0.027; Fig. 2B). A similar trend was also seen for the expression of *MUC1* mRNA, although not significant (*P* = 0.069; Fig. 2B). On the contrary, mid-level expression of *MUC2* mRNA (*P* = 0.038) associated with worse survival, whereas a trend for low- and high-level expression of *MUC13* mRNA towards an unfavorable outcome was noted (*P* = 0.068; Fig. 2B). However, only a significant association between high-level *MUC13* expression and worse survival was defined by the Cox-proportional hazards model (*P* = 0.016; Fig. 2B). In addition, the gastric cardia (*P* = 0.02) and stage 3 (*P* = 0.001) also correlated with a worse outcome (Fig. 2B).

## Microbiome dysbiosis alters between the different mucin phenotype groups

**Fig. 3:**
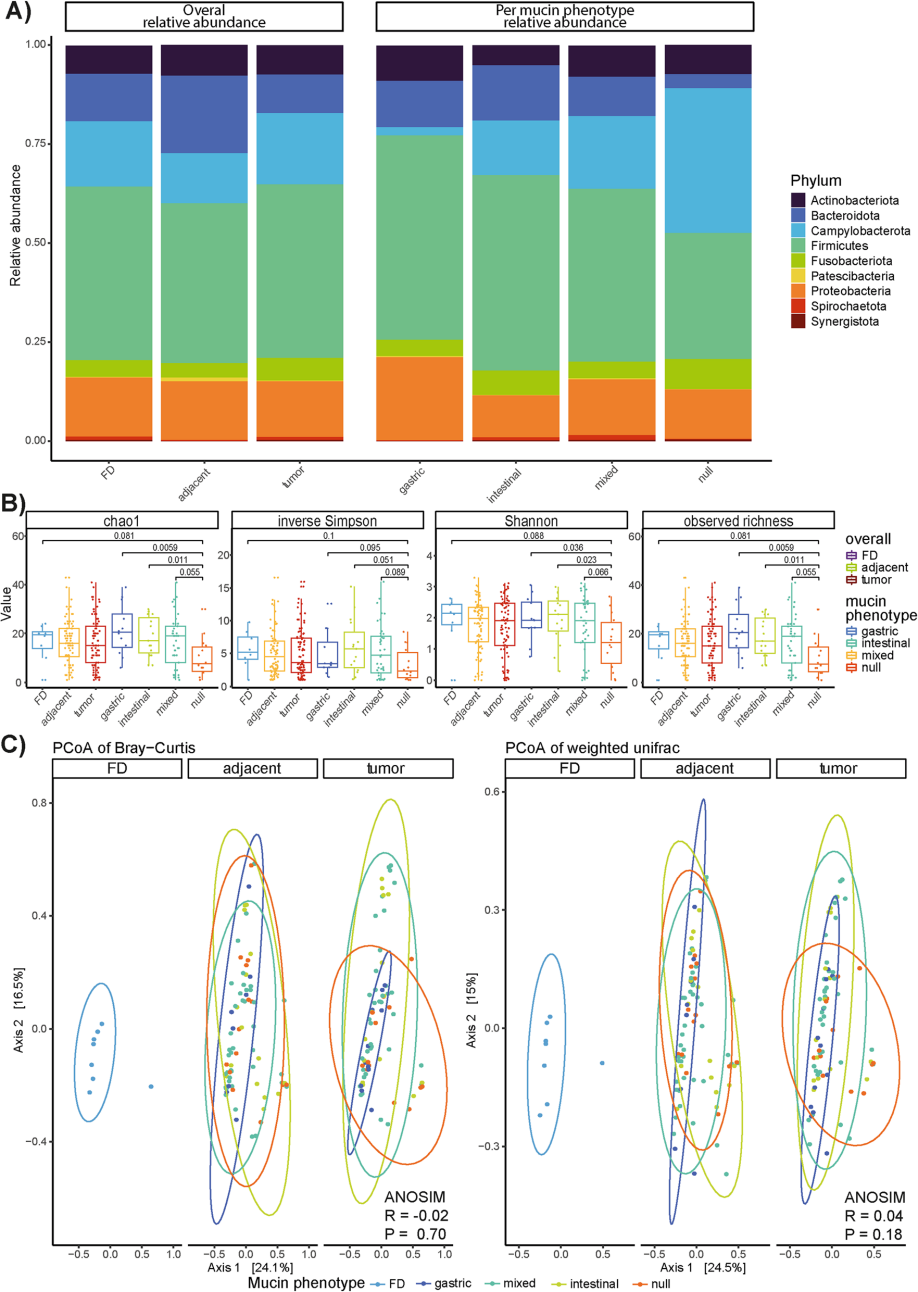
Differences in microbiota composition between FD, tumor, and adjacent non-tumor tissues and between tumors with gastric, intestinal, mixed, and null mucin phenotypes. **A** Mean relative abundance of phyla in all tissue types (FD, tumor, and adjacent non-tumor samples; *n* = 8, 83, and 80, respectively) and in each mucin phenotype group assigned to the gastric tumor samples (gastric, intestinal, mixed, and null; *n* = 12, 15, 41, and 14, respectively). **B** Boxplots of four common alpha-diversity indices (i.e., chao1, inverse Simpson, observed richness, and Shannon index) for FD, tumor, and adjacent non-tumor tissues (*n* = 8, 83, and 80, respectively) as well as for tumors with gastric, intestinal, mixed, and null mucin phenotypes (*n* = 12, 15, 41, and 14, respectively). **C** PCoA using the Bray–Curtis (left) and weighted unifrac (right) distance measures for FD, tumor, and adjacent non-tumor tissue samples (*n* = 8, 83, and 80, respectively). The points were colored according to the mucin phenotype assigned to each tumor. The percentage of variance captured by the axes and the 95% confidence intervals per mucin phenotype (drawn as ellipses) are also shown

To determine dysbiosis associated with the different GC mucin phenotypes, we first evaluated the alterations in microbiome structure at the phylum level using the relative bacterial abundance per tissue type (tumor, adjacent non-tumor, and FD tissues) and per mucin phenotype (Fig. 3A). Overall, significant differences in abundance for *Patescibacteria* were found between (1) FD and adjacent non-tumor tissues (*P* = 0.024), (2) FD and tumor tissues (*P* = 0.014), and (3) FD tissues and tumors with intestinal (*P* = 0.057), mixed (*P* = 0.068), and null (0.0191) mucin phenotypes (Fig. 3A). Significant differences in abundance were also noted for *Campylobacterota* between tumors with a null mucin phenotype and gastric (*P* = 0.0126) or intestinal (*P* = 0.0295) mucin phenotype which can be assigned to the differential abundance of the *Helicobacter* genus among the phenotypes. Additionally, *Bacteroidota* differed significantly in abundance between tumors with a null mucin phenotype and those with a gastric (*P* = 0.0067), intestinal (*P* = 0.0032), or mixed (*P* = 0.0249) mucin phenotype (Fig. 3A). This latter phylum was also significantly altered between the FD cases and tumors with a null mucin phenotype (*P* = 0.0079; Fig. 3A).

In support of the above, changes in community composition were further investigated using alpha (i.e., within samples) and beta (i.e., between samples) diversity measures at the genus level (Fig. 3B, C). Tumor samples with a null mucin phenotype showed a significantly lower alpha diversity compared to those with an intestinal mucin phenotype based on all four indexes investigated and with a mixed mucin phenotype based on the Chao1 and observed richness only (Fig. 3B). When considering the observed richness and Shannon indexes, a significant decrease in alpha diversity of tumors with a null mucin phenotype compared to those with a gastric mucin phenotype was also noted (Fig. 3B). Beta diversity was analyzed using the Bray–Curtis and weighted UniFrac phylogenetic distance metrics and visualized in Principal Coordinate Analysis (PCoA) plots (Fig. 3C). The first two axes captured 24.1% and 16.5% for the Bray–Curtis distances and was similar to the results for the weighted UniFrac distances (24.5% and 15%). Interestingly, tumor samples with an intestinal mucin phenotype were more spread over axis 2 while tumors with a gastric phenotype remained strongly clustered around the FD samples. Contrary, when testing for differences in community composition using ANOSIM, no significant differences were found (Fig. 3C).

**Fig. 4:**
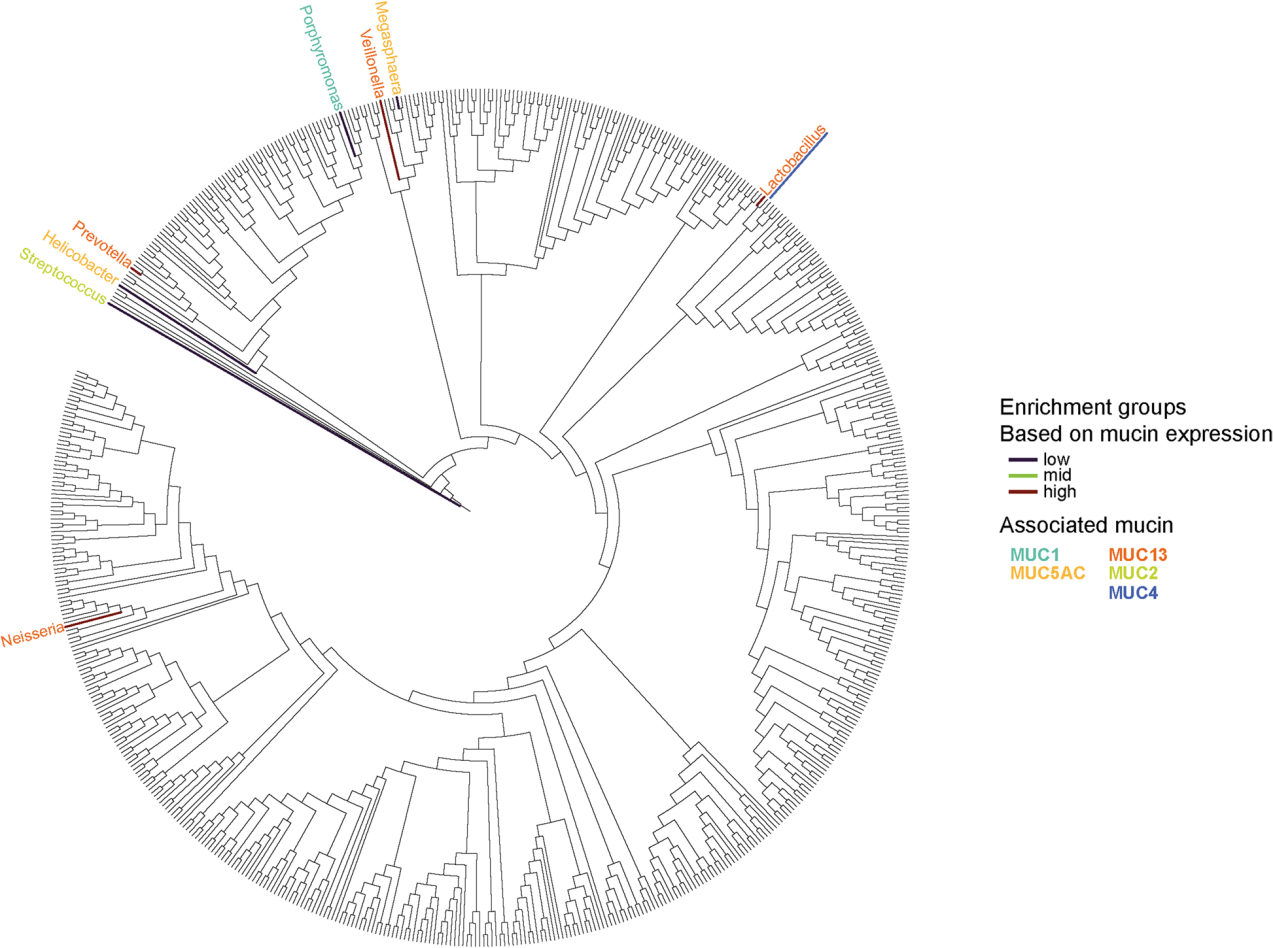
Phylogenetic tree of GC-enriched and GC-depleted bacteria associated with aberrant mucin expression. Genera that are significantly abundant or underrepresented in gastric tumors (*n* = 82) with mid, low, or high mRNA expression of a gastric (MUC1, MUC5AC, MUC6) or intestinal (MUC2, MUC4, MUC13) mucin are shown by color. The colored branches highlight the mucin mRNA expression levels (low, mid, or high) whereas the corresponding-colored genera represent the mucins they associate with. In case of overlapping mucin associations, the color of the underlining indicates the second mucin association

To identify the bacterial taxa that are differentially present in GC compared to FD cases, an ALDEx2 analysis was conducted. Overall, the genera *Veillonella, Porphyromonas*, and *Prevotella* were found to be depleted in GC compared to the FD group whereas the abundance of *Corynebacterium*, *Fusobacterium, Streptococcus Porphyromonas*, and *Prevotella* differed significantly between paired tumor and non-tumor tissues (Fig. S2). To further investigate the influence of aberrant mucin expression on bacterial enrichment or depletion in gastric tumors, differential abundance analysis and Spearman’s correlation tests were performed assessing the association between bacterial genera and individual mucins or mucin phenotypes. The *Helicobacter* genus was found to be enriched in tumors with a null mucin phenotype compared to the other phenotypes (Fig. S3) and in tumor samples with low *MUC5AC *(Fig. 4; Fig. S5) expression. Also, *Megasphaera* was enriched in samples with low *MUC5AC* expression (Fig. 4; Fig. S5). In gastric adenocarcinomas with low *MUC1* mRNA levels, an abundance of *Porphyromonas* was observed (Fig. 4; Fig. S4). Regarding associations between bacterial genera and intestinal mucin expression, depletion in *Streptococcus* was identified in gastric adenocarcinomas with high *MUC2* mRNA levels (Fig. 4; Fig. S6) and *Lactobacillus* was significantly more abundant in tumors with mid-level *MUC4* mRNA expression (Fig. 4; Fig. S7). Interestingly, *Lactobacillus*, *Neisseria, Prevotella*, and *Veillonella* were enriched in gastric tumor samples with high *MUC13* expression (Fig. 4; Fig. S8). These latter genera also significantly correlated with *MUC13* mRNA levels as was also seen for *Helicobacter* (Fig. S1)*.* Furthermore, *Lactobacillus* and *Neisseria* positively correlated with *MUC4* and *MUC2* mRNA expression, respectively (Fig. S1), whereas *Porphyromonas* and *Corynebacterium* significantly associated with the gastric mucin expression levels (Fig. S1).

### Distinct community complexity in gastric adenocarcinoma with different mucin phenotypes

**Fig. 5:**
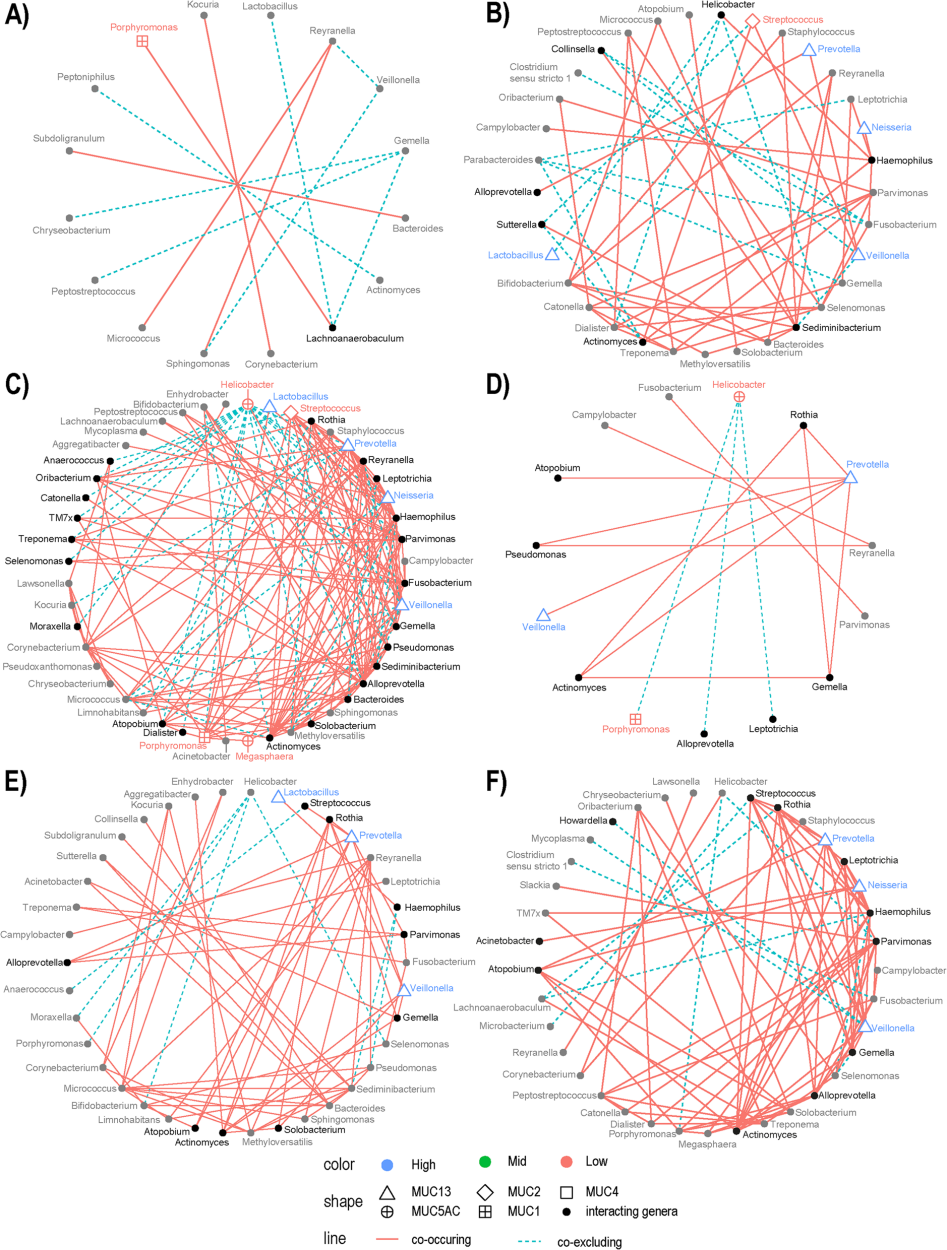
Correlation of GC-enriched and GC-depleted genera associated with a mucin phenotype or aberrant MUC13 expression. Each sphere represents the bacterial network of gastric adenocarcinomas with **A** gastric mucin phenotype (*n* = 12), **B** intestinal mucin phenotype (*n* = 15), **C** mixed mucin phenotype (*n* = 41), **D** null mucin phenotype (*n* = 14), **E** low MUC13 mRNA expression (*n* = 26), and **F** high MUC13 mRNA expression (*n* = 34). Each genus found to participate in the bacterial community is represented by a point and connected through lines with interacting bacterial taxa. The red lines are positive associations between bacterial genera while the blue dotted lines are negative interactions. The point shape represents the association between a genus and a specific mucin (i.e., the genus is found to be differentially abundant with different expression levels of the mucin). The color of the point (+ genus name) represents the mucin mRNA expression level in which the genus was found to be enriched. If the genus was not found to be differentially abundant with different mucin expression levels, the shape is a full circle that is either gray (does not directly interact with other genera associated with a specific mucin) or black (the genus interacts directly with a mucin-associated bacterial taxa)

Co-occurrence and co-excluding interactions were analyzed using the probabilistic model of species co-occurrence to estimate positive and negative associations among bacterial genera in GC [42]. Figure 5 shows the bacterial networks identified in the different mucin phenotype groups and gastric tumors with low and high *MUC13* expression. The overall number of interactions differed significantly between the different mucin phenotype groups (Pearson’s *χ*^2^, *P* < 0.0001), with most interactions seen in tumors with mixed and intestinal mucin phenotypes (Fig. 5B, C; Table S4). The same pattern was also seen for the number of positive and negative associations (Pearson’s *χ*^2^, *P* < 0.0001). Furthermore, the ratio of co-excluding to co-occurring interactions differed between the different mucin phenotypes with relatively more co-occurring interactions in tumors with an intestinal, mixed, and null mucin phenotype (negative to positive ratio: 0.23, 0.30, and 0.25, respectively; Fig. 5B–D) while those with a gastric mucin phenotype had more co-excluding than co-occurring interactions (1.4; Fig. 5A). In addition, a Pearson’s *χ*^2^ test was done at genus level to test for differences in contribution to the network of different genera between gastric tumors assigned to the different mucin phenotypes. The residuals (i.e., the difference between the expected value and the observed one) were used as a measure of correlation (i.e., a high residual means a bigger contribution of the genus to a significant statistical test), and only those greater than two were considered. In adenocarcinomas with a gastric mucin phenotype, *Lachnoanaerobaculum*, *Gemella*, and *Reyranella* had proportionally more interactions compared to tumors with another mucin phenotype (Fig. 5A; Table S5). *Selenomonas* and *Treponema* had proportionally more influence on the bacterial community of samples with an intestinal mucin phenotype, while *Rothia* and *Prevotella* had a higher contribution in tumor samples with a null mucin phenotype (Fig. 5; Table S5). In gastric adenocarcinoma with an intestinal mucin phenotype, most interactions were seen for *Parvimonas, Sediminibacterium, Helicobacter, Selenomonas, Fusobacterium, Reyranella, Treponema, Leptotrichia, Haemophilus, Neisseria, Veillonella, Prevotella*, and *Streptococcus* (Fig. 5B; Table S5). Bacterial genera with the most interactions in tumor samples with a mixed mucin phenotype were *Alloprevotella, Fusobacterium, Sediminibacterium, Neisseria, Veillonella, Reyranella, Rothia, Streptococcus, Haemophilus, Parvimonas, Prevotella*, and* Helicobacter* (Fig. 5C; Table S5), whereas *Rothia, Prevotella*, and* Helicobacter* contributed more in samples with a null mucin phenotype (Fig. 5D; Table S5).

Finally, *Veillonella*, *Neisseria*, and *Prevotella* were found to play a role in shaping the community structure in tumors with high *MUC13* expression (Fig. 5E, F; Table S6).

### Enrichment of oral and intestinal microbes in GC depends on the mucin phenotype

To investigate whether oral and intestinal bacteria have a preference for a certain mucin phenotype in gastric tumors, we determined the overall distribution of oral and intestinal genera by profiling the sequences of all samples against the Human Oral Microbiome and the Human Intestinal 16S rRNA gene reference databases [40, 41]. The microbiome of the FD cohort showed a trend of enrichment in oral microbial taxa compared to the tumor and tumor-adjacent microbiome (*P* = 0.10; Fig. S9). When comparing the tumor samples with different mucin phenotypes to the FD cohort, a significant depletion in oral taxa is seen in the null mucin phenotype (*P* = 0.018) and a trend towards depletion is also seen in tumors with a mixed mucin phenotype (*P* = 0.12; Fig. S9). Additionally, when considering the tumor samples only, a trend in oral microbial enrichment was seen in samples with an intestinal mucin phenotype compared to the null mucin phenotype (*P* = 0.1; Fig. S9). Inversely, for the intestinal microbial species, a significant enrichment was seen in tumor samples with a null mucin phenotype compared to those with a gastric (*P* = 0.027) and intestinal (*P* = 0.02) mucin phenotype. Of note, a similar trend was also seen between the null and mixed mucin phenotype tumors (*P* = 0.064; Fig. S9).

### Microbiome functional capacity in GC is defined by the mucin phenotype

**Fig. 6:**
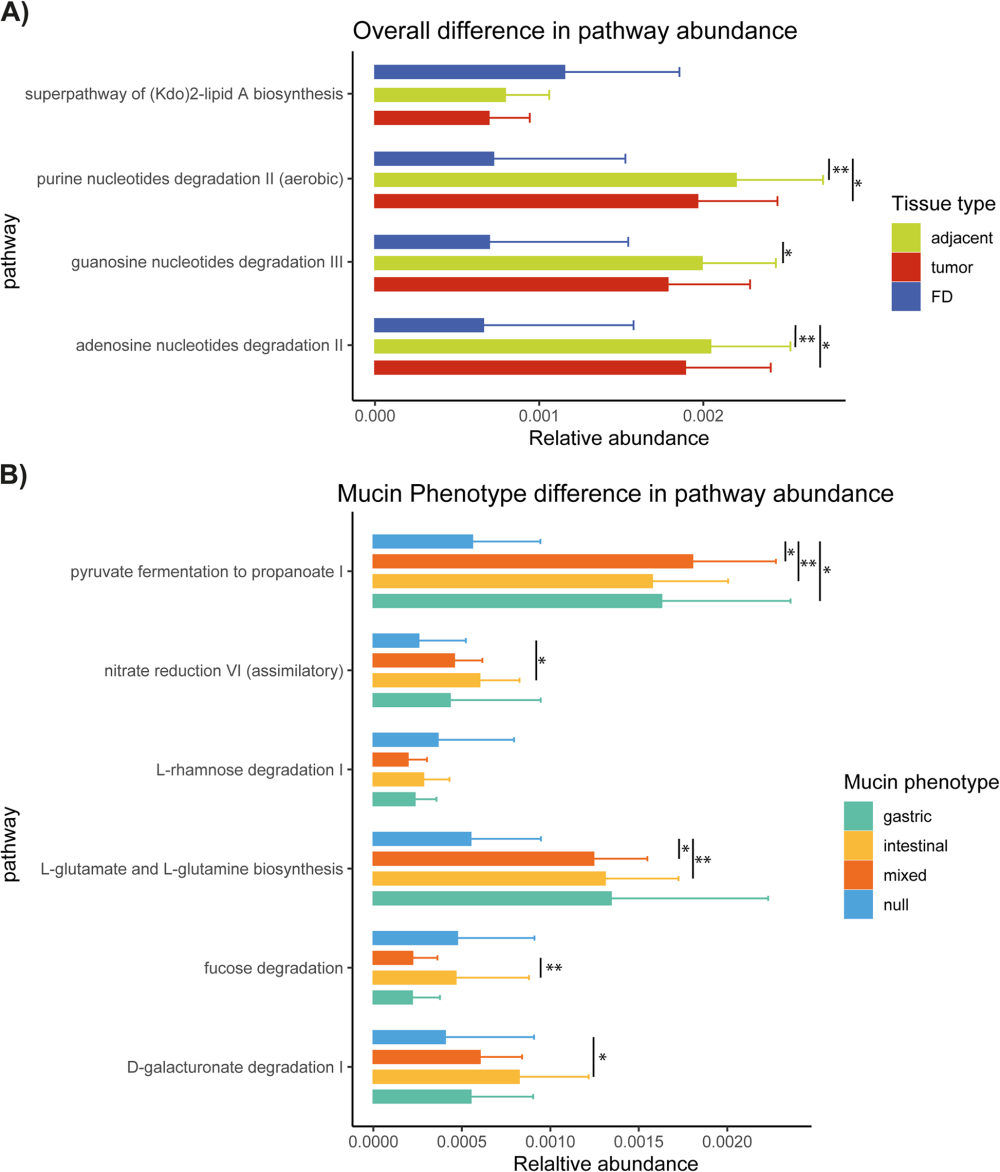
Relative abundance of metagenomic pathways is defined by the mucin phenotype present in gastric adenocarcinomas. Functional classification of the predicted metagenome content of the microbiota of FD, tumor, and adjacent non-tumor samples (*n* = 8, 83, and 80, respectively) (**A**) and of gastric adenocarcinomas with gastric, intestinal, mixed, or null mucin phenotypes (*n* = 12, 15, 41, and 14, respectively) (**B**) using PICRUSt2. Significance was considered for *P* < 0.05 and indicated by *P < 0.05 and **P < 0.01

The functional capacity of the mucosa-associated microbiome for each sample type (FD, tumor, and adjacent non-tumor tissues) and mucin phenotype or aberrantly expressed mucin in gastric tumors was estimated through metagenomic inference using PICRUSt2. Differences in relative pathway abundance were tested using ALDEx2. When considering the FD, tumor, and adjacent non-tumor samples, three pathways, i.e., purine, guanosine, and adenosine nucleotide degradation, were found to be differentially abundant and enriched in the GC cohort (Fig. 6A). Furthermore, five predicted KEGG pathways were found to be differentially represented between the tumors assigned to different mucin phenotypes with most of the pathways being depleted in tumors with a null mucin phenotype except for the fucose degradation pathway (Fig. 6B).

This assessment was repeated for each mucin and is summarized in Table S7. For the *MUC1* and *MUC2* mRNA expression levels*,* no differentially abundant pathways were found. Interestingly, in samples with low *MUC6* and *MUC5AC* expression, the purine ribonucleosides degradation pathway was depleted. In contrast, an enrichment of sugar degrading pathways was seen in samples with a high *MUC13* expression (i.e., sucrose degradation IV (sucrose phosphorylase), glycogen degradation I (bacterial), and galactose degradation I (Leloir pathway)). Additionally, heterolactic fermentation was also enriched in high *MUC13* expressing samples and pyruvate fermentation to propanoate I was depleted in the samples with a normal *MUC13* expression compared to the high and low levels. An enrichment of the sucrose degradation IV (sucrose phosphorylase) pathway was also seen in samples with high *MUC2* expression. The abovementioned changes in the metabolic potential of the gastric cancer microbiome suggest a high reliance on mucins as a food source.

## Discussion

It is generally well-accepted that microbial dysbiosis is a dynamic process correlating with the progression to gastric cancer [7]. Previous studies always considered the whole stomach as one habitat while alterations in gastric mucosal microbiota across different stomach microhabitats (i.e., tumoral and peritumoral microhabitats) also occurs [7]. Therefore, adjacent non-tumor tissues should also be taken into account as premalignant gastric lesions (i.e., atrophy and intestinal metaplasia) can occur revealing cancer-related features such as mucin and microbial signatures with predictive potential for malignant transformation [46]. Furthermore, changes described in microbial composition due to the overrepresentation of arising new taxa remain inconsistent in gastric adenocarcinomas [7, 47]. This discrepancy may be due to tumor microenvironment heterogeneity with variable expression of gastric and intestinal mucins shaping the microbiota community and influencing disease outcome. In this study, we assigned gastric adenocarcinomas to four different phenotypes based on mucin expression, with the intestinal mucin phenotype being significantly associated with a worse survival rate. This finding was further substantiated by high-level *MUC13* expression which also correlated with an unfavorable outcome, highlighting a key role for this intestinal mucin in gastric carcinogenesis. MUC13 overexpression has previously been described in GC and such aberrant *MUC13* signaling is known to protect colorectal cancer cells from death via NF-kB pathway activation thereby impacting on therapeutic efficacy and disease outcome [12, 48]. Whether the upregulation of MUC13 has similar capacities to inhibit gastric tumor cell death requires further investigation. In addition, decreased expression levels of *MUC1*, *MUC5AC*, and *MUC6* also associated with a poorer outcome, highlighting the importance of gastric mucins in GC to increase the chances of survival [19, 49–51].

In terms of the composition of the gastric microbiota, distinct dysbiosis in gastric adenocarcinomas compared to our FD cohort and along with differences based on mucin phenotype groups was observed, with the known gastric phyla being widely present, including *Campylobacterota* [52, 53]. This latter phylum, previously part of the *Proteobacteria,* mainly consisted of *Helicobacter* genus members in both our tumor and FD samples. This shows that the FD group was colonized with *Helicobacter*, despite no gastric lesions being found, further highlighting that this genus is a core member of the normal gastric microbiota as determined previously [7]. Significant differences in *Helicobacter* abundance was noted among the different mucin phenotype groups, with the highest presence found in tumors with a null mucin phenotype [54]. This can be explained by a decrease in gastric mucin expression, a characteristic of these adenocarcinomas. More specifically, MUC5AC provides an important adherence site for *H. pylori*, but its absence can facilitate colonization whereas MUC6 has antibiotic properties against *H. pylori* [55].

Reduced microbial diversity is an overall major feature in many disease states, including inflammatory bowel diseases and cancer [53, 56]. In our different patient cohorts, the lowest alpha diversity was also seen in gastric adenocarcinomas compared to FD and adjacent non-tumor tissues, and more specifically in tumors with a null mucin phenotype possibly due to a higher *Helicobacter* abundance. In contrast, no differences in beta diversity were found which could in part be due to different confounding factors influencing microbiome composition, such as age, gender, diet, and drug use [57, 58]. More specifically, microbial diversity changes throughout the human life span and is known to be associated with gender whereas diet and the usage of drugs induce temporary shifts in gut microbiota composition [59–61].

Interestingly, from our genus/species level classification, we observed that several taxa, including *Corynebacterium*, *Fusobacterium*, *Streptoccus*, *Porphyromonas, Veillonella*, and *Prevotella,* significantly differed in abundance between tumor and adjacent non-tumor or FD tissues. These abovementioned genera have already been linked to gastrointestinal cancers [5, 6, 62–65]. When investigating the GC microbiome in more detail, other bacterial genera were also found to be associated with aberrant mucin expression in the tumor. More specifically, *Lactobacillus* was found to be significantly more abundant in adenocarcinomas with high *MUC4* and *MUC13* expression. Members of this genus normally reside in the intestinal mucosa but are capable of colonizing and proliferating in the cancerous stomach [5, 20, 66] and most probably in the presence of an intestinal mucin environment. Furthermore, *Prevotella, Veillonella*, and *Neisseria* seemed also to have a higher affinity for tumors with *MUC13* overexpression which are in fact inhabitants of the oral cavity but can be opportunistic pathogens [6, 52, 53, 67]. As oral microbiota were also enriched in tumors with an intestinal mucin phenotype, a shift from predominantly gastric to intestinal mucins could thus affect the abundance of pro-inflammatory oral microbes in intestinal mucin phenotype tumors, specifically those with *MUC13* overexpression. Whether these oral bacteria play a role in MUC13-driven gastric carcinogenesis warrants further investigation. Importantly, we also identified the oral taxa *Porphyromonas* and *Megasphaera* to be enriched in adenocarcinoma with low *MUC1* and *MUC5AC* expression, respectively. Both genera have been associated with worse survival in GI cancers, further underlining the favorable role of gastric mucin abundance in GC outcomes [68].

Not only individual bacterial drivers, but also the microbial networks that reside within define the disease-specific microenvironment [4, 69]. As such, we observed the overall highest co-occurring and co-excluding interactions of enriched and depleted bacteria in tumors with a mixed and intestinal mucin phenotype. Several bacterial genera were identified to play a role in shaping the microbial community. In particular, the potential role of oral pathogenic taxa in GC is highlighted by the observed centralities of *Lachnoanaerobaculum*, *Gemella*, and* Reyranella *in gastric mucin phenotype tumors; *Rothia *and *Prevotella* in null mucin phenotype tumors; and *Neisseria*, *Veillonella*, and *Prevotella* in both intestinal and mixed mucin phenotype tumors and *Fusobacterium* [70]. Although *Fusobacterium* is not differentially abundant with regard to mucin expression or phenotype, this genus is known to be an important player in gastrointestinal cancers [71–73].

After having analyzed the diversity and composition of the gastric microbiota in relation to aberrant mucin expression in GC, we finally addressed the functional features of the GC microbiota which can affect host metabolism [6]. We demonstrated an overall decrease in metabolic activity in tumors with a null mucin phenotype which could be explained by a decline in bacterial community complexity. When investigating metabolic changes between tumors with different mucin expression levels, we observed predicted functional shifts in short-chain fatty acid (SCFA) fermentation, amino acid, and sugar degradation that may reflect compositional differences in mucin expression in the tumor microenvironment. Specifically, the changes in carbohydrate digestion are predictive of bacterial production of SCFAs, which have been linked to the hyperproliferation of cells in gastrointestinal cancers [6]. Furthermore, bacterial SCFAs have also been shown to stimulate intestinal mucin expression, suggesting their importance in intestinal mucin phenotype tumors [20].

## Conclusions

Taken together, our study identified distinct mucin-microbiome signatures shaping the tumor microenvironment in gastric cancer, with an intestinal or aberrant MUC13 mucin environment associated with a poor outcome. We also showed that members of the oral pathogenic taxa, such as *Neisseria, Prevotella, *and* Veillonella*, are potential drivers in MUC13-mediated signaling in GC which could be useful biomarkers in predicting disease outcomes. As not all pre-malignant gastric conditions will eventually evolve into cancer, such oral taxa-MUC13 signatures in patients with pre-cancerous stadia could also help predict the potential to further evolve into cancer. Furthermore, adenocarcinomas with an intestinal mucin phenotype do favor the establishment of pro-inflammatory oral bacteria, forming strong co-occurrence networks. Ultimately, understanding these mucin-microbiome signatures in gastric carcinogenesis may impact GC prevention and treatment strategies and adequate independent external validation in other GC cohorts is therefore recommended. Additionally, future research must also consider the inclusion of healthy patients as controls, since it currently remains unclear how microbiota composition relates to FD [74]. Nevertheless, this is the first study implicating mucins in both dysbiosis and disease outcomes in gastric cancer.

## Supplementary Information

**Additional file 1: Figure S1.** Associations of mucin mRNA expression with bacterial abundance and clinical data of GC patients. Correlogram of GC (*n*=108) patients. Spearman’s rank order correlation values (r) are shown from blue (–1.0) to red (1.0); r values are indicated by color. *P* values are indicated by black asterisks (*<0.05; **<0.01; ***<0.001). The considered parameters are age, gender (*n*= 108), tumor stage (*n*= 101), Lauren’s classification (*n*=106), survival (i.e. deceased or alive after 5 years follow-up; n= 67 and 30; respectively), MUC1 (*n*= 100), MUC5AC (*n*= 100), MUC6 (*n*=100), MUC2 (*n*= 100), MUC4 (*n*= 99) and MUC13 (n= 98) mRNA expression.

**Additional file 2: Figure S2.** Relative bacterial abundance of the genera found to be differentially abundant between control, tumor adjacent and tumor tissue. The relative abundance of each bacterial genus found to be differentially abundant between control and paired (highlighted by grey dashed lines) tumor and adjacent non-tumor tissues (*n*= 8, 83 and 80; respectively) using ALDEx2 is shown. *P*-values found to be significant are shown on the plots and were calculated using a Wilcoxon rank sum test.

**Additional file 3: Figure S3.** Relative bacterial abundance of the genera found to be differentially abundant between tumor tissues having different mucin phenotypes. The relative abundance of each bacterial genus found to be differentially abundant between tumor tissues having different mucin phenotypes (gastric, intestinal, mixed and null; *n*= 10, 15, 41 and 14; respectively) using ALDEx2 are shown. *P*-values found to be significant are shown on the plots and were calculated using a Wilcoxon rank sum test.

**Additional file 4: Figure S4.** Relative bacterial abundance of the genera found to be differentially abundant between tumor tissues having a high or low MUC1 mRNA expression. The relative abundance of each bacterial genus found to be differentially abundant between tumor samples having a high or low MUC1 mRNA expression level (*n*= 38 and 41; respectively) using ALDEx2 is shown. *P*-values found to be significant are shown on the plots and calculated using a Wilcoxon rank sum test.

**Additional file 5: Figure S5.** Relative bacterial abundance of the genera found to be differentially abundant between tumor tissues having a high or low MUC5AC mRNA expression. The relative abundance of each bacterial genus found to be differentially abundant between tumor samples having a high or low MUC5AC mRNA expression level (*n*= 38 and 41; respectively) using ALDEx2 is shown. *P*-values found to be significant are shown on the plots and calculated using a Wilcoxon rank sum test.

**Additional file 6: Figure S6.** Relative bacterial abundance of the genera found to be differentially abundant between tumor tissues having a high, mid or low MUC2 mRNA expression. The relative abundance of each bacterial genus found to be differentially abundant between tumor samples having a high, mid or low MUC2 mRNA expression level (*n*= 26, 9, 47; respectively) using ALDEx2 is shown. *P*-values found to be significant are shown on the plots and calculated using a Wilcoxon rank sum test.

**Additional file 7: Figure S7.** Relative bacterial abundance of the genera found to be differentially abundant between tumor tissues having a high, mid or low MUC4 mRNA expression. The relative abundance of each bacterial genus found to be differentially abundant between tumor samples having a high, mid or low MUC4 mRNA expression level (*n*= 28, 7, 47; respectively) using ALDEx2 is shown. *P*-values found to be significant are shown on the plots and calculated using a Wilcoxon rank sum test.

**Additional file 8: Figure S8.** Relative bacterial abundance of the genera found to be differentially abundant between tumor tissues having a high, mid or low MUC13 mRNA expression. The relative abundance of each bacterial genus found to be differentially abundant between tumor samples having a high, mid or low MUC13 mRNA expression level (*n*= 34, 22, 26; respectively) using ALDEx2 is shown. *P*-values found to be significant are shown on the plots and calculated using a Wilcoxon rank sum test.

**Additional file 9: Figure S9.** Pooled relative abundance of oral and intestinal bacterial species for the FD, tumor and non-tumor adjacent samples and the tumor tissues further divided according to their mucin phenotype. The relative abundance of ASV’s classified up to species level using the HOM and HIT databases were pooled per sample, according to their preferred habitat as detailed in the HOM-database, into either being part of the oral or intestinal microbiome. The relative abundances are plotted for the control, tumor and non-tumor adjacent tissues (*n*= 8, 83 and 80; respectively). For the tumor tissues, the relative abundance of the oral and intestinal species was also shown according to the respective mucin phenotype of the tumor tissue (gastric, intestinal, mixed and null; *n*= 10, 15, 41 and 14; respectively). Significant differences between control, tumor and non-tumor adjacent tissues and gastric adenocarcinomas with different mucin phenotypes are shown on the plots and calculated using a Wilcoxon rank sum test.

**Additional file 10: Table S1.** demographic information of the included patients. **Table S2.** overview of the used QuantiTect primers. **Table S3.** 90% confidence interval of the relative mucin mRNA expression levels of functional dyspepsia patients. **Table S4.** number of interactions within the bacterial communities of samples with different mucin phenotypes. **Table S5.** Analysis of interactions in bacterial communities associated with different mucin phenotypes. **Table S6.** Analysis of interactions in bacterial communities associated with different expression level of MUC13. **Table S7.** Differentially abundant metagenomic pathways.

## Data Availability

The raw 16S rRNA sequencing data that support the findings of this study are available from the NCBI Sequence Read Archive (BioProject: PRJNA924141). All R markdown files used for the data analysis of the paper can be found on GitHub (BaptisteO/MucinMicrobiomeGC). All other data supporting the findings of this study are available within the article and its supplementary information files.

## Abbreviations

ANOSIM: Analysis of similarities
ASV: Amplicon sequence variants
CNRQ: Calibrated normalized relative quantities
CI: Confidence interval
EC: Enzyme commission
FD: Functional dyspepsia
GC: Gastric cancer
HOM: Human oral microbiome
*H. pylori*: *Helicobacter pylori*
IHC: Immunohistochemistry
LL: Lower limit
MUC: Mucin
PCoA: Principal coordinate analysis
SPEM: Spasmolytic polypeptide expressing metaplasia
SCFA: Short-chain fatty acid
UL: Upper limit
