# Supplementary figures and images for: Mucin-microbiome signatures shape the tumor microenvironment in gastric cancer
Source: Microbiome. 2023 Apr 21;11:86. doi: 10.1186/s40168-023-01534-w (PMC10120190; doi:10.1186/s40168-023-01534-w)

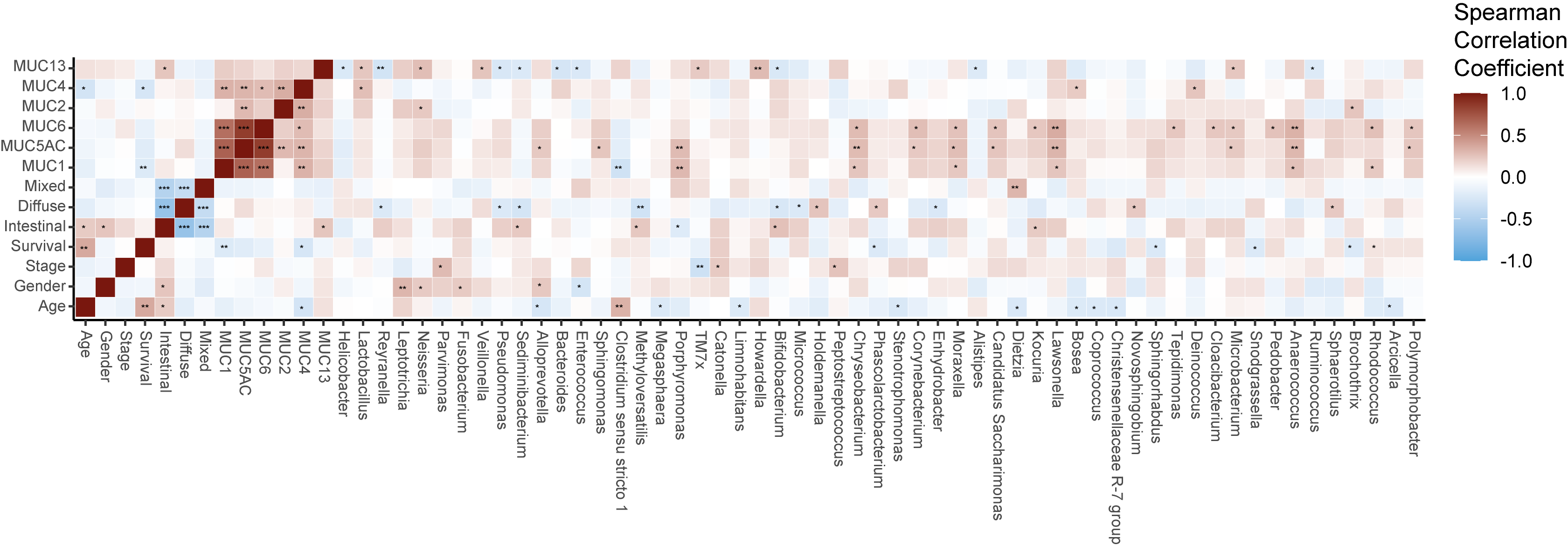

Supplement: Supplementary file 2 — Additional file 1: Figure S1. Associations of mucin mRNA expression with bacterial abundance and clinical data of GC patients. Correlogram of GC (n=108) patients. Spearman’s rank order correlation values (r) are shown from blue (–1.0) to red (1.0); r values are indicated by color. P values are indicated by black asterisks (*<0.05; **<0.01; ***<0.001). The considered parameters are age, gender (n= 108), tumor stage (n= 101), Lauren’s classification (n=106), survival (i.e. deceased or alive after 5 years follow-up; n= 67 and 30; respectively), MUC1 (n= 100), MUC5AC (n= 100), MUC6 (n=100), MUC2 (n= 100), MUC4 (n= 99) and MUC13 (n= 98) mRNA expression. [file 40168_2023_1534_MOESM1_ESM.tif]

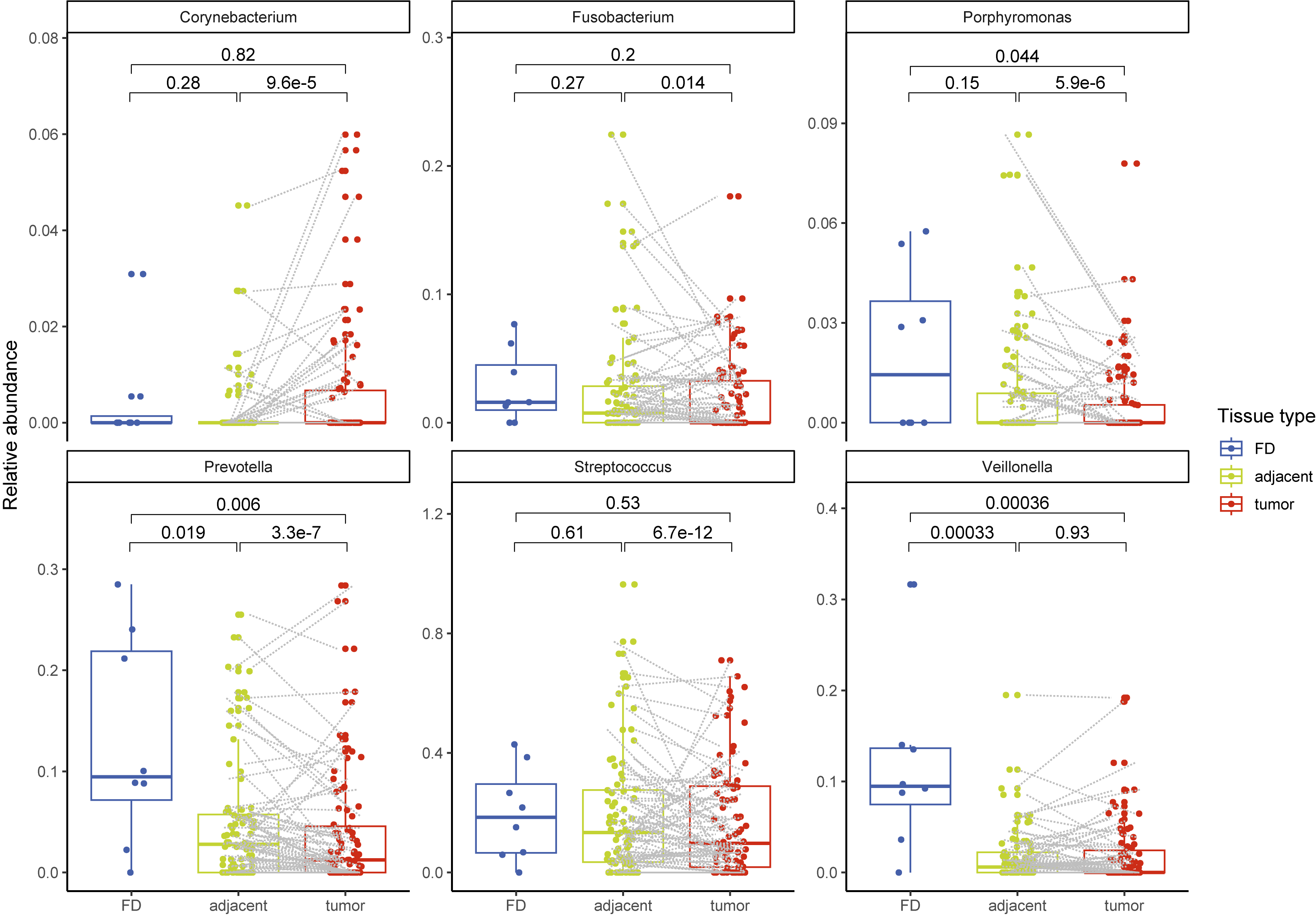

Supplement: Supplementary file 3 — Additional file 2: Figure S2. Relative bacterial abundance of the genera found to be differentially abundant between control, tumor adjacent and tumor tissue. The relative abundance of each bacterial genus found to be differentially abundant between control and paired (highlighted by grey dashed lines) tumor and adjacent non-tumor tissues (n= 8, 83 and 80; respectively) using ALDEx2 is shown. P-values found to be significant are shown on the plots and were calculated using a Wilcoxon rank sum test. [file 40168_2023_1534_MOESM2_ESM.tif]

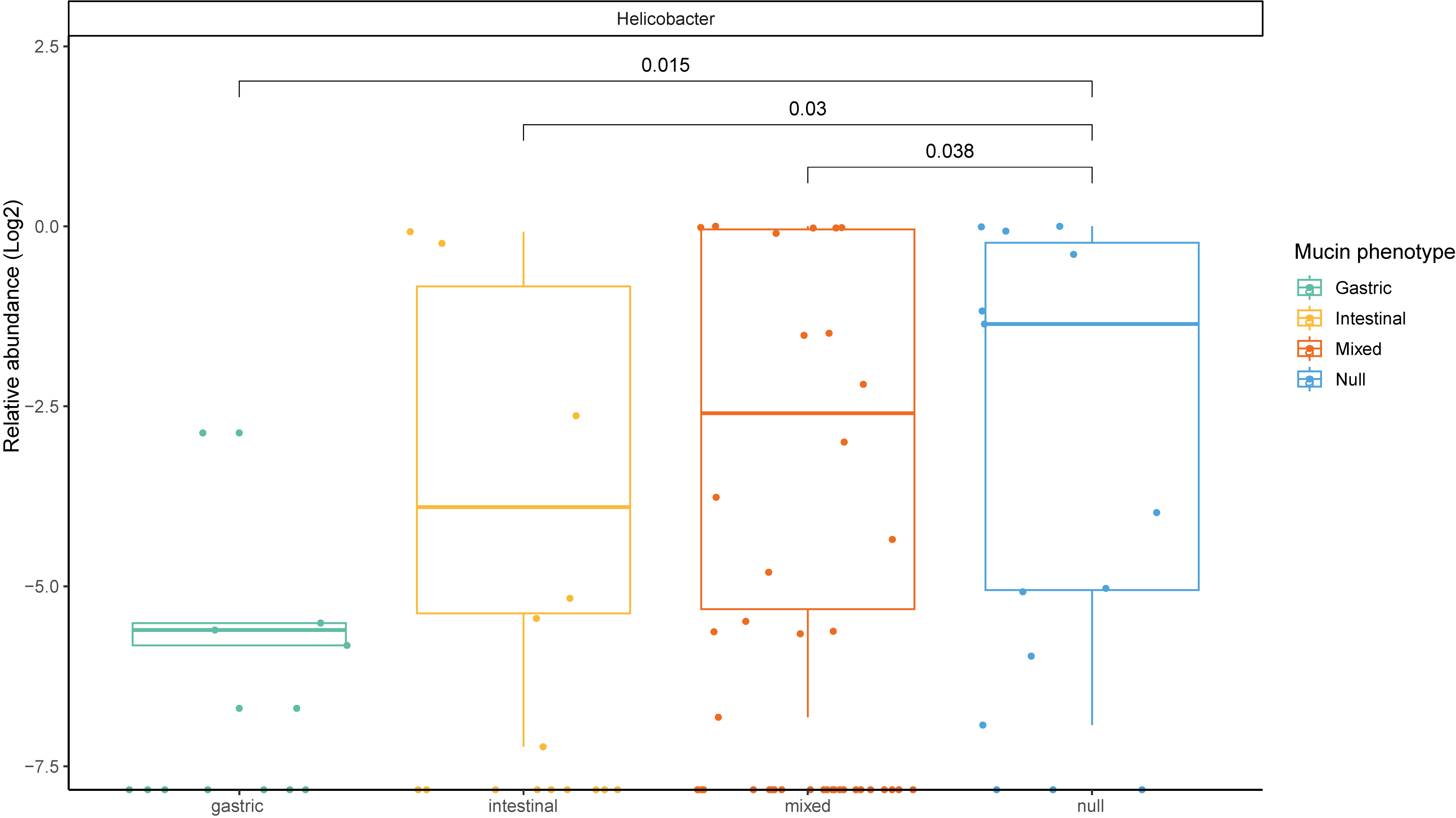

Supplement: Supplementary file 4 — Additional file 3: Figure S3. Relative bacterial abundance of the genera found to be differentially abundant between tumor tissues having different mucin phenotypes. The relative abundance of each bacterial genus found to be differentially abundant between tumor tissues having different mucin phenotypes (gastric, intestinal, mixed and null; n= 10, 15, 41 and 14; respectively) using ALDEx2 are shown. P-values found to be significant are shown on the plots and were calculated using a Wilcoxon rank sum test. [file 40168_2023_1534_MOESM3_ESM.tif]

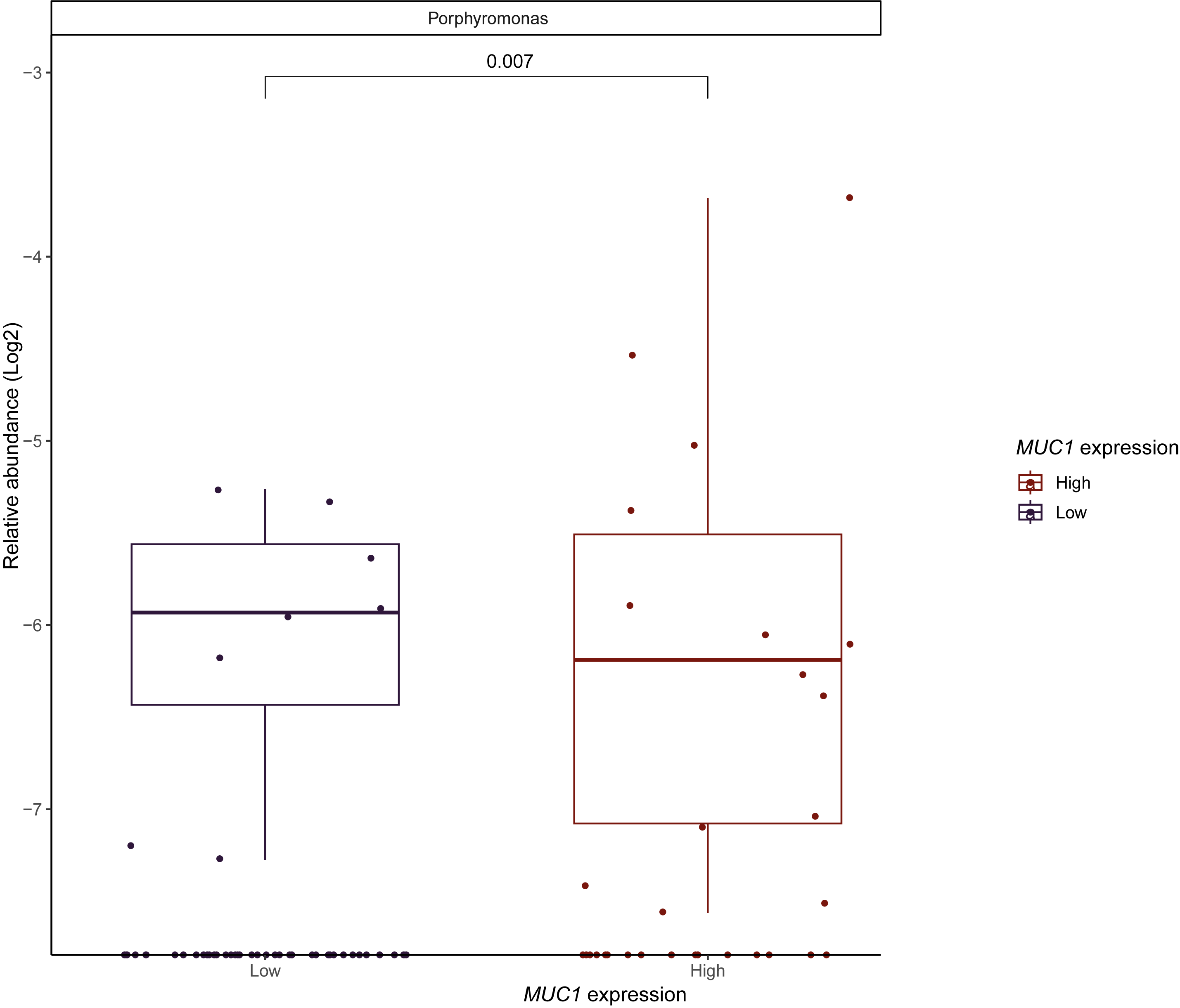

Supplement: Supplementary file 5 — Additional file 4: Figure S4. Relative bacterial abundance of the genera found to be differentially abundant between tumor tissues having a high or low MUC1 mRNA expression. The relative abundance of each bacterial genus found to be differentially abundant between tumor samples having a high or low MUC1 mRNA expression level (n= 38 and 41; respectively) using ALDEx2 is shown. P-values found to be significant are shown on the plots and calculated using a Wilcoxon rank sum test. [file 40168_2023_1534_MOESM4_ESM.tif]

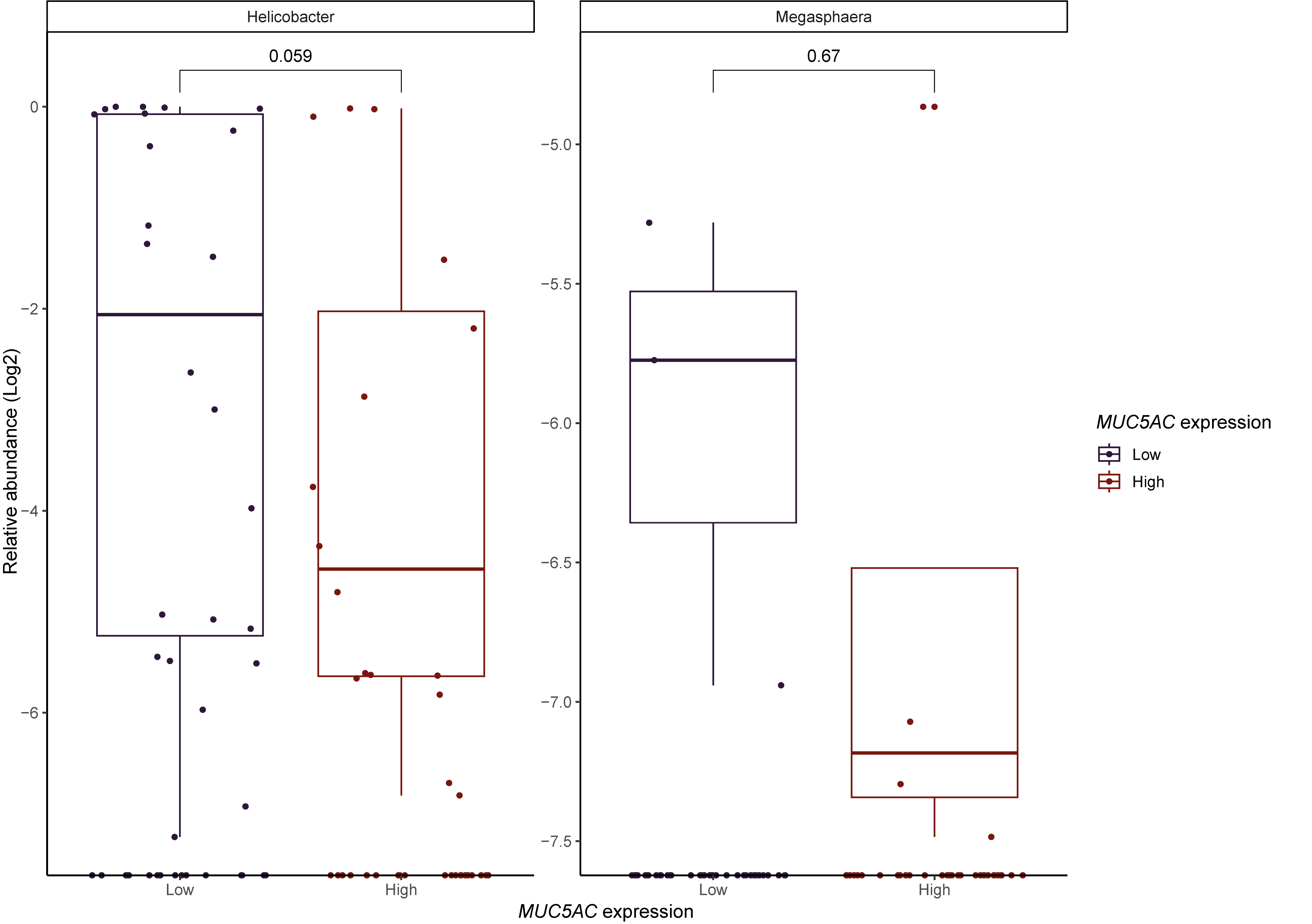

Supplement: Supplementary file 6 — Additional file 5: Figure S5. Relative bacterial abundance of the genera found to be differentially abundant between tumor tissues having a high or low MUC5AC mRNA expression. The relative abundance of each bacterial genus found to be differentially abundant between tumor samples having a high or low MUC5AC mRNA expression level (n= 38 and 41; respectively) using ALDEx2 is shown. P-values found to be significant are shown on the plots and calculated using a Wilcoxon rank sum test. [file 40168_2023_1534_MOESM5_ESM.tif]

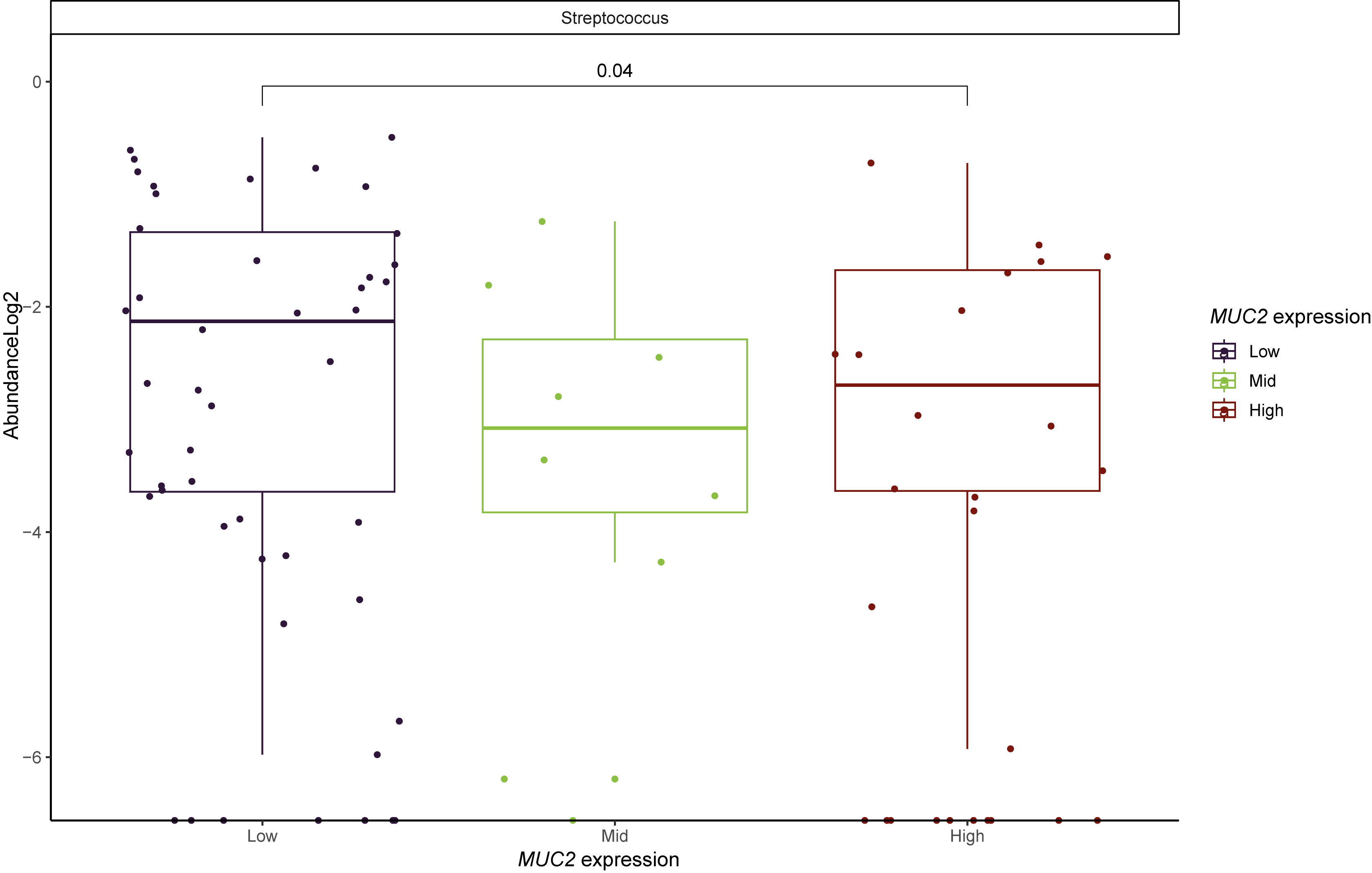

Supplement: Supplementary file 7 — Additional file 6: Figure S6. Relative bacterial abundance of the genera found to be differentially abundant between tumor tissues having a high, mid or low MUC2 mRNA expression. The relative abundance of each bacterial genus found to be differentially abundant between tumor samples having a high, mid or low MUC2 mRNA expression level (n= 26, 9, 47; respectively) using ALDEx2 is shown. P-values found to be significant are shown on the plots and calculated using a Wilcoxon rank sum test. [file 40168_2023_1534_MOESM6_ESM.tif]

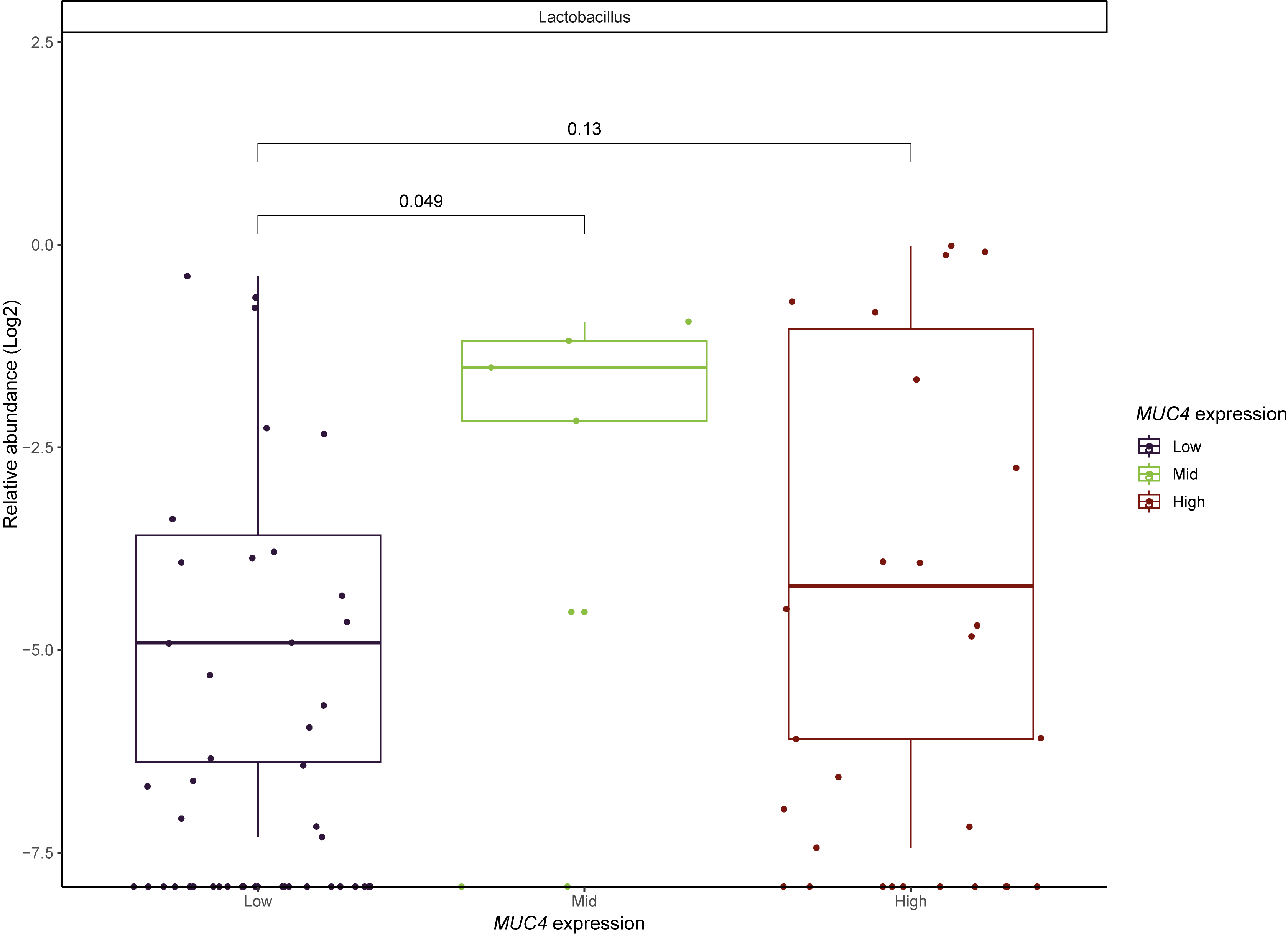

Supplement: Supplementary file 8 — Additional file 7: Figure S7. Relative bacterial abundance of the genera found to be differentially abundant between tumor tissues having a high, mid or low MUC4 mRNA expression. The relative abundance of each bacterial genus found to be differentially abundant between tumor samples having a high, mid or low MUC4 mRNA expression level (n= 28, 7, 47; respectively) using ALDEx2 is shown. P-values found to be significant are shown on the plots and calculated using a Wilcoxon rank sum test. [file 40168_2023_1534_MOESM7_ESM.tif]

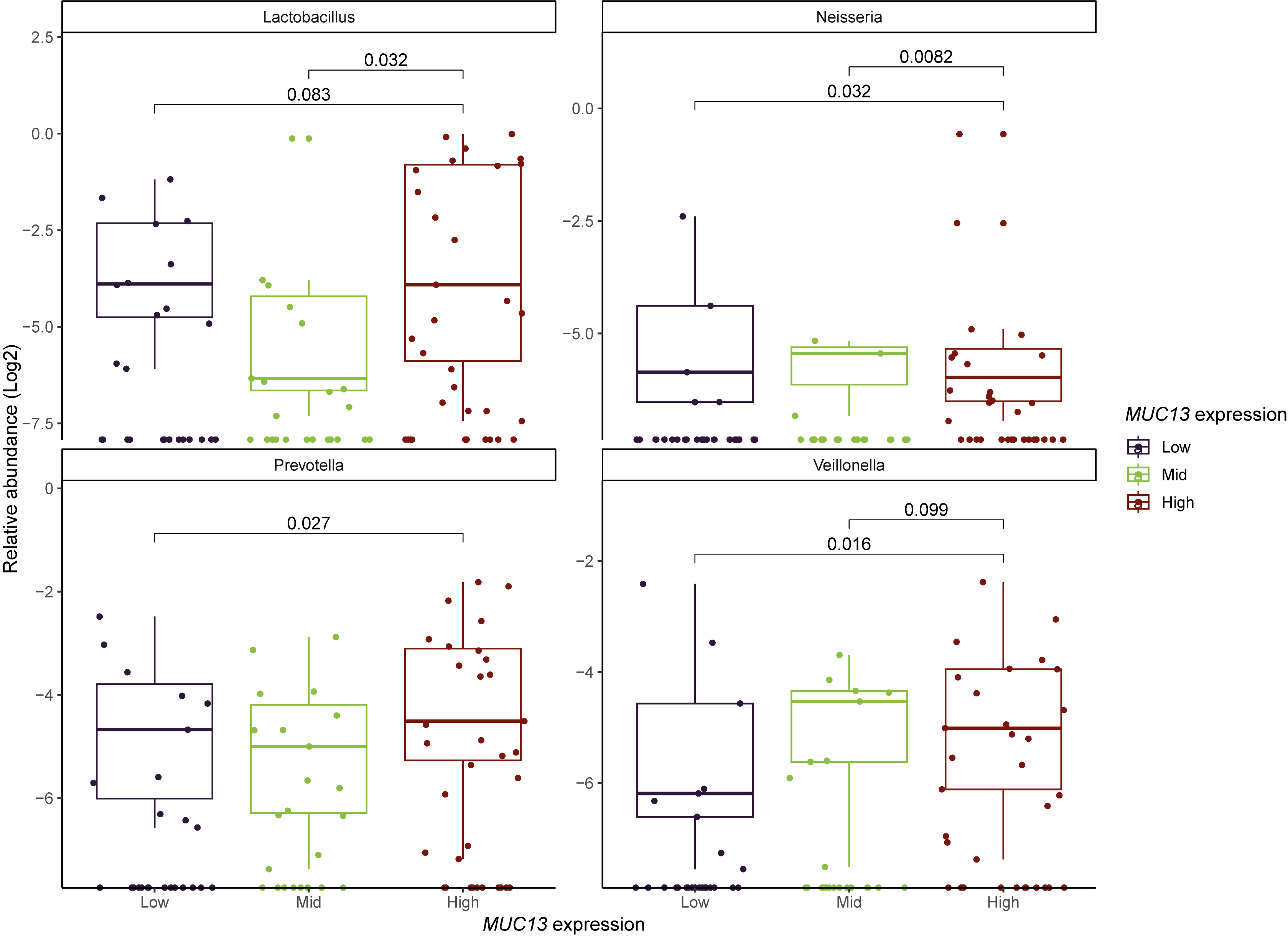

Supplement: Supplementary file 9 — Additional file 8: Figure S8. Relative bacterial abundance of the genera found to be differentially abundant between tumor tissues having a high, mid or low MUC13 mRNA expression. The relative abundance of each bacterial genus found to be differentially abundant between tumor samples having a high, mid or low MUC13 mRNA expression level (n= 34, 22, 26; respectively) using ALDEx2 is shown. P-values found to be significant are shown on the plots and calculated using a Wilcoxon rank sum test. [file 40168_2023_1534_MOESM8_ESM.tif]

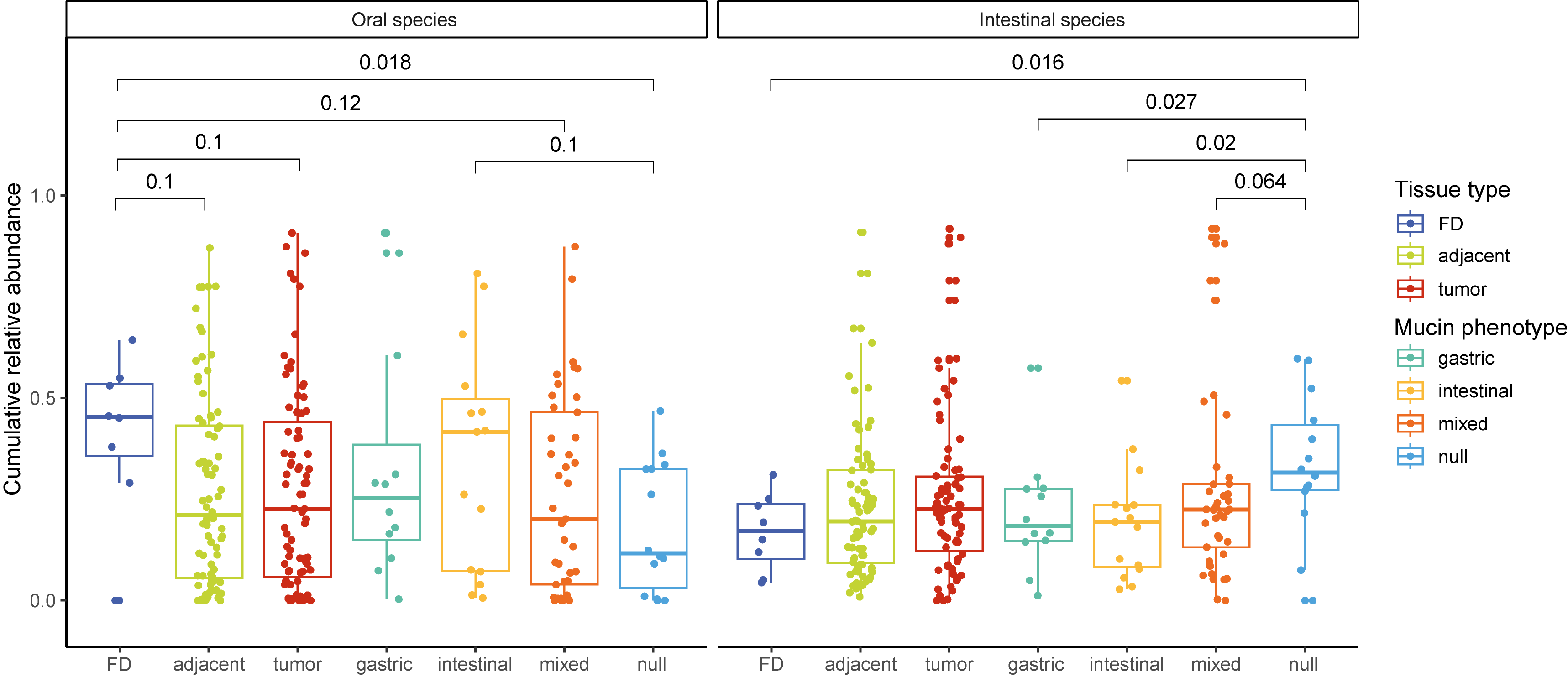

Supplement: Supplementary file 10 — Additional file 9: Figure S9. Pooled relative abundance of oral and intestinal bacterial species for the FD, tumor and non-tumor adjacent samples and the tumor tissues further divided according to their mucin phenotype. The relative abundance of ASV’s classified up to species level using the HOM and HIT databases were pooled per sample, according to their preferred habitat as detailed in the HOM-database, into either being part of the oral or intestinal microbiome. The relative abundances are plotted for the control, tumor and non-tumor adjacent tissues (n= 8, 83 and 80; respectively). For the tumor tissues, the relative abundance of the oral and intestinal species was also shown according to the respective mucin phenotype of the tumor tissue (gastric, intestinal, mixed and null; n= 10, 15, 41 and 14; respectively). Significant differences between control, tumor and non-tumor adjacent tissues and gastric adenocarcinomas with different mucin phenotypes are shown on the plots and calculated using a Wilcoxon rank sum test. [file 40168_2023_1534_MOESM9_ESM.tif]
