# Supplementary material for: Mucin-microbiome signatures shape the tumor microenvironment in gastric cancer
Source: Microbiome. 2023 Apr 21;11:86. doi: 10.1186/s40168-023-01534-w (PMC10120190; doi:10.1186/s40168-023-01534-w)
Supplement: Supplementary file 11 — Additional file 10: Table S1. demographic information of the included patients. Table S2. overview of the used QuantiTect primers. Table S3. 90% confidence interval of the relative mucin mRNA expression levels of functional dyspepsia patients. Table S4. number of interactions within the bacterial communities of samples with different mucin phenotypes. Table S5. Analysis of interactions in bacterial communities associated with different mucin phenotypes. Table S6. Analysis of interactions in bacterial communities associated with different expression level of MUC13. Table S7. Differentially abundant metagenomic pathways. [file 40168_2023_1534_MOESM10_ESM.docx]

**Supplementary Tables**

## Table S1: demographic information of the included patients

| **Parameter** | **Gastric cancer (n = 108), n (%)** | | **Functional dyspepsia (n=20)** |
| --- | --- | --- | --- |
| **Age (years; mean ± SD)** |  | |  |
| Male  Female | 67.07 ± 12.5  66.48 ± 12.9 | | 44.83 ± 18.8  45.55 ± 19.1 |
| **Gender** |  |  |  |
| Male | 72 (66.7) |  | 7 (35) |
| Female | 36 (33.3) |  | 13 (65) |
| **Origin** |  |  |  |
| Lithuania | 43 (39.8) |  | 0 |
| Ghent | 48 (44.4) |  | 0 |
| Antwerp | 17 (15.7) |  | 20 (100) |
| **Tumour location** |  |  |  |
| Cardia | 31 (28.7) |  |  |
| Fundus | 1 (0.9) |  |  |
| Corpus | 41 (38) |  |  |
| Antrum | 27 (25) |  |  |
| Linitis plastica | 5 (4.6) |  |  |
| Missing | 3 (2.8) |  |  |
| **Lauren classification** |  |  |  |
| Diffuse | 35 (32.4) |  |  |
| Intestinal | 54 (50.0) |  |  |
| Mixed | 17 (15.7) |  |  |
| Missing | 2 (1.9) |  |  |
| **5-year survival** |  |  |  |
| Alive | 30 (27.8) |  |  |
| Dead | 67 (62.0) |  |  |
| Missing | 10 (9.2) |  |  |
| **Primary tumour (T)** |  |  |  |
| T1 | 16 (14.8) |  |  |
| T2 | 40 (37.0) |  |  |
| T3 | 35 (32.4) |  |  |
| T4 | 15 (13.9) |  |  |
| Missing | 2 (1.9) |  |  |
| **Lymph nodes (N)** |  |  |  |
| NX | 2 (1.9) |  |  |
| N0 | 36 (33.3) |  |  |
| N1 | 22 (20.4) |  |  |
| N2 | 28 (25.9) |  |  |
| N3 | 18 (16.6) |  |  |
| Missing | 2 (1.9) |  |  |
| **Distant metastasis (M)** |  |  |  |
| MX | 26 (24.1) |  |  |
| M0 | 55 (50.9) |  |  |
| M1 | 22 (20.4) |  |  |
| Missing | 5 (4.6) |  |  |
| **Tumour grade (G)** |  |  |  |
| G1 | 12 (11.1) |  |  |
| G2 | 31 (28.7) |  |  |
| G3 | 49 (45.4) |  |  |
| G4 | 12 (11.1) |  |  |
| Missing | 4 (3.7) |  |  |
| **Tumour stage** |  |  |  |
| I | 10 (9.3) |  |  |
| II | 13 (12.0) |  |  |
| III | 23 (21.3) |  |  |
| IV | 55 (50.9) |  |  |
| Missing | 7 (6.5) |  |  |

## Table S2: overview of the used QuantiTect primers

| **Gene ID** | **Primer** |
| --- | --- |
| MUC1 | QuantiTect Primer Assay QT00015379 |
| MUC5AC | QuantiTect Primer Assay QT00088991 |
| MUC6 | QuantiTect Primer Assay QT00237839 |
| MUC2 | QuantiTect Primer Assay QT01004675 |
| MUC4 | QuantiTect Primer Assay QT00045479 |
| MUC13 | QuantiTect Primer Assay QT00002478 |
| GAPDH | QuantiTect Primer Assay QT00079247 |
| β-actin | QuantiTect Primer Assay QT00095431 |

## Table S3: 90% confidence interval of the relative mucin mRNA expression levels of functional dyspepsia patients.

|  | Lower bound  90% CI | Upper bound  90%CI |
| --- | --- | --- |
| **MUC1** | 0,95 | 1,25 |
| **MUC5AC** | 0,90 | 1,45 |
| **MUC6** | 1,02 | 1,67 |
| **MUC2** | 1,06 | 1,74 |
| **MUC4** | 1,04 | 1,86 |
| **MUC13** | 1,01 | 2,17 |

## Table S4: number of interactions within the bacterial communities of samples with different mucin phenotypes.

|  | **Gastric** | **Intestinal** | **Mixed** | **Null** | **χ²** |
| --- | --- | --- | --- | --- | --- |
| co-occurring | 5 | 43 | 141 | 12 | < 2.2E-16 |
| co-excluding | 7 | 13 | 33 | 3 | < 2.82E-8 |
| total | 12 | 56 | 174 | 15 | < 2.2E-16 |

## Table S5: Analysis of interactions in bacterial communities associated with different mucin phenotypes

|  | Number of interactions | | | | Residuals | | | |
| --- | --- | --- | --- | --- | --- | --- | --- | --- |
| **Genus** | **Gastric** | **Intestinal** | **Mixed** | **Null** | **Gastric** | **Intestinal** | **Mixed** | **Null** |
| Lachnoanaerobaculum | 1 | 0 | 0 | 0 | 4,41 | -0,47 | -0,82 | -0,24 |
| Catonella | 0 | 1 | 0 | 0 | -0,22 | 1,68 | -0,82 | -0,24 |
| Bifidobacterium | 0 | 1 | 0 | 0 | -0,22 | 1,68 | -0,82 | -0,24 |
| Treponema | 0 | 3 | 0 | 0 | -0,37 | 2,90 | -1,43 | -0,42 |
| Selenomonas | 0 | 4 | 0 | 0 | -0,43 | 3,35 | -1,65 | -0,48 |
| Micrococcus | 0 | 0 | 1 | 0 | -0,22 | -0,47 | 0,39 | -0,24 |
| Kocuria | 0 | 0 | 1 | 0 | -0,22 | -0,47 | 0,39 | -0,24 |
| Campylobacter | 0 | 0 | 1 | 0 | -0,22 | -0,47 | 0,39 | -0,24 |
| Atopobium | 0 | 0 | 1 | 0 | -0,22 | -0,47 | 0,39 | -0,24 |
| Megasphaera | 0 | 0 | 1 | 0 | -0,22 | -0,47 | 0,39 | -0,24 |
| Limnohabitans | 0 | 0 | 1 | 0 | -0,22 | -0,47 | 0,39 | -0,24 |
| Pseudoxanthomonas | 0 | 0 | 1 | 0 | -0,22 | -0,47 | 0,39 | -0,24 |
| Lawsonella | 0 | 0 | 1 | 0 | -0,22 | -0,47 | 0,39 | -0,24 |
| Dialister | 0 | 2 | 1 | 0 | -0,37 | 1,67 | -0,72 | -0,42 |
| Sphingomonas | 0 | 0 | 2 | 0 | -0,31 | -0,66 | 0,56 | -0,34 |
| Chryseobacterium | 0 | 0 | 2 | 0 | -0,31 | -0,66 | 0,56 | -0,34 |
| Porphyromonas | 0 | 0 | 2 | 0 | -0,31 | -0,66 | 0,56 | -0,34 |
| Acinetobacter | 0 | 0 | 2 | 0 | -0,31 | -0,66 | 0,56 | -0,34 |
| Moraxella | 0 | 0 | 2 | 0 | -0,31 | -0,66 | 0,56 | -0,34 |
| Methyloversatilis | 0 | 1 | 2 | 0 | -0,37 | 0,43 | -0,02 | -0,42 |
| Lactobacillus | 1 | 0 | 3 | 0 | 1,88 | -0,93 | 0,18 | -0,48 |
| Solobacterium | 0 | 1 | 3 | 0 | -0,43 | 0,14 | 0,18 | -0,48 |
| Actinomyces | 1 | 2 | 3 | 0 | 1,36 | 0,61 | -0,53 | -0,59 |
| Corynebacterium | 1 | 0 | 4 | 0 | 1,59 | -1,04 | 0,33 | -0,54 |
| Bacteroides | 1 | 2 | 4 | 0 | 1,18 | 0,38 | -0,34 | -0,64 |
| Pseudomonas | 0 | 0 | 5 | 0 | -0,48 | -1,04 | 0,88 | -0,54 |
| Staphylococcus | 0 | 2 | 5 | 0 | -0,57 | 0,38 | 0,12 | -0,64 |
| Leptotrichia | 0 | 3 | 6 | 0 | -0,65 | 0,74 | -0,04 | -0,72 |
| Alloprevotella | 0 | 0 | 7 | 0 | -0,57 | -1,24 | 1,04 | -0,64 |
| Fusobacterium | 0 | 4 | 7 | 0 | -0,72 | 1,04 | -0,16 | -0,80 |
| Sediminibacterium | 0 | 5 | 7 | 0 | -0,75 | 1,48 | -0,39 | -0,84 |
| Neisseria | 0 | 1 | 8 | 0 | -0,65 | -0,69 | 0,77 | -0,72 |
| Veillonella | 1 | 2 | 9 | 0 | 0,59 | -0,38 | 0,31 | -0,84 |
| Streptococcus | 0 | 3 | 10 | 0 | -0,78 | 0,10 | 0,40 | -0,87 |
| Haemophilus | 0 | 3 | 10 | 0 | -0,78 | 0,10 | 0,40 | -0,87 |
| Gemella | 3 | 2 | 2 | 1 | 4,30 | 0,19 | -1,47 | 0,78 |
| Parvimonas | 0 | 5 | 10 | 1 | -0,86 | 0,81 | -0,25 | 0,07 |
| Reyranella | 3 | 3 | 9 | 2 | 2,48 | -0,37 | -0,74 | 1,01 |
| Rothia | 0 | 0 | 10 | 3 | -0,78 | -1,68 | 0,40 | 2,57 |
| Helicobacter | 0 | 4 | 18 | 3 | -1,08 | -0,62 | 0,26 | 1,28 |
| Prevotella | 0 | 2 | 13 | 5 | -0,97 | -1,13 | -0,15 | 3,55 |

## Table S6: Analysis of interactions in bacterial communities associated with different expression level of *MUC13*

|  | number of interactions | | residuals | |
| --- | --- | --- | --- | --- |
| **Genus** | **low MUC13** | **high MUC13** | **low MUC13** | **high MUC13** |
| Reyranella | 7 | 0 | 2,20 | -1,97 |
| Sediminibacterium | 7 | 0 | 2,20 | -1,97 |
| Bacteroides | 5 | 0 | 1,86 | -1,67 |
| Pseudomonas | 3 | 0 | 1,44 | -1,29 |
| Methyloversatilis | 3 | 0 | 1,44 | -1,29 |
| Micrococcus | 3 | 0 | 1,44 | -1,29 |
| Lactobacillus | 1 | 0 | 0,83 | -0,75 |
| Sphingomonas | 1 | 0 | 0,83 | -0,75 |
| Atopobium | 1 | 0 | 0,83 | -0,75 |
| Limnohabitans | 1 | 0 | 0,83 | -0,75 |
| Bifidobacterium | 1 | 0 | 0,83 | -0,75 |
| Corynebacterium | 2 | 1 | 0,58 | -0,52 |
| Helicobacter | 4 | 3 | 0,50 | -0,45 |
| Gemella | 2 | 2 | 0,17 | -0,15 |
| Actinomyces | 1 | 1 | 0,12 | -0,11 |
| Prevotella | 5 | 6 | 0,05 | -0,04 |
| Rothia | 6 | 8 | -0,09 | 0,08 |
| Selenomonas | 1 | 2 | -0,29 | 0,26 |
| Veillonella | 3 | 5 | -0,29 | 0,26 |
| Fusobacterium | 2 | 4 | -0,41 | 0,37 |
| Porphyromonas | 0 | 1 | -0,67 | 0,60 |
| Campylobacter | 0 | 1 | -0,67 | 0,60 |
| Staphylococcus | 0 | 1 | -0,67 | 0,60 |
| Megasphaera | 0 | 1 | -0,67 | 0,60 |
| Catonella | 0 | 1 | -0,67 | 0,60 |
| Peptostreptococcus | 0 | 1 | -0,67 | 0,60 |
| Parvimonas | 3 | 8 | -0,85 | 0,76 |
| Dialister | 0 | 2 | -0,94 | 0,84 |
| Streptococcus | 2 | 7 | -1,00 | 0,89 |
| Haemophilus | 2 | 7 | -1,00 | 0,89 |
| Leptotrichia | 1 | 5 | -1,02 | 0,91 |
| Solobacterium | 1 | 5 | -1,02 | 0,91 |
| Alloprevotella | 0 | 3 | -1,15 | 1,03 |
| Treponema | 0 | 5 | -1,49 | 1,33 |
| Neisseria | 0 | 5 | -1,49 | 1,33 |

## Table S7: Differentially abundant metagenomic pathways

| **pathway** | **description** | **P** |
| --- | --- | --- |
| **FD - non-tumor adjacent - tumor** | | |
| KDO-NAGLIPASYN-PWY | superpathway of (Kdo)2-lipid A biosynthesis | 4,61E-02 |
| PWY-6353 | purine nucleotides degradation II (aerobic) | 2,27E-02 |
| PWY-6608 | guanosine nucleotides degradation III | 2,89E-02 |
| SALVADEHYPOX-PWY | adenosine nucleotides degradation II | 2,13E-02 |
| **mucin phenotype** | | |
| FUCCAT-PWY | fucose degradation | 4,08E-02 |
| GALACTUROCAT-PWY | D-galacturonate degradation I | 1,74E-02 |
| P108-PWY | pyruvate fermentation to propanoate I | 6,02E-03 |
| PWY-5505 | L-glutamate and L-glutamine biosynthesis | 9,22E-03 |
| PWY490-3 | nitrate reduction VI (assimilatory) | 2,12E-02 |
| RHAMCAT-PWY | L-rhamnose degradation I | 3,47E-02 |
| ***MUC5AC* expression levels** | | |
| ARO-PWY | chorismate biosynthesis I | 4,03E-02 |
| CALVIN-PWY | Calvin-Benson-Bassham cycle | 4,84E-02 |
| COMPLETE-ARO-PWY | superpathway of aromatic amino acid biosynthesis | 4,17E-02 |
| GLUCONEO-PWY | gluconeogenesis I | 4,12E-02 |
| ILEUSYN-PWY | L-isoleucine biosynthesis I (from threonine) | 3,43E-02 |
| NONOXIPENT-PWY | pentose phosphate pathway (non-oxidative branch) | 3,74E-02 |
| P4-PWY | superpathway of L-lysine, L-threonine and L-methionine biosynthesis I | 4,89E-02 |
| PWY-3001 | superpathway of L-isoleucine biosynthesis I | 3,96E-02 |
| PWY-5104 | L-isoleucine biosynthesis IV | 1,16E-02 |
| PWY-6163 | chorismate biosynthesis from 3-dehydroquinate | 3,98E-02 |
| PWY-6545 | pyrimidine deoxyribonucleotides de novo biosynthesis III | 2,60E-02 |
| PWY-6700 | queuosine biosynthesis | 3,90E-02 |
| PWY-6897 | thiamin salvage II | 4,91E-02 |
| PWY-7371 | 1,4-dihydroxy-6-naphthoate biosynthesis II | 4,14E-02 |
| PWY0-1296 | purine ribonucleosides degradation | 2,85E-02 |
| SER-GLYSYN-PWY | superpathway of L-serine and glycine biosynthesis I | 3,22E-02 |
| VALSYN-PWY | L-valine biosynthesis | 3,44E-02 |
| ***MUC6* expression levels** | | |
| 1CMET2-PWY | N10-formyl-tetrahydrofolate biosynthesis | 1,38E-01 |
| ANAEROFRUCAT-PWY | homolactic fermentation | 1,82E-01 |
| ANAGLYCOLYSIS-PWY | glycolysis III (from glucose) | 1,82E-01 |
| ARO-PWY | chorismate biosynthesis I | 1,83E-01 |
| ASPASN-PWY | superpathway of L-aspartate and L-asparagine biosynthesis | 1,82E-01 |
| BRANCHED-CHAIN-AA-SYN-PWY | superpathway of branched amino acid biosynthesis | 9,76E-02 |
| CALVIN-PWY | Calvin-Benson-Bassham cycle | 1,82E-01 |
| COA-PWY | coenzyme A biosynthesis I | 1,82E-01 |
| COMPLETE-ARO-PWY | superpathway of aromatic amino acid biosynthesis | 1,83E-01 |
| DENOVOPURINE2-PWY | superpathway of purine nucleotides de novo biosynthesis II | 9,75E-02 |
| DTDPRHAMSYN-PWY | dTDP-L-rhamnose biosynthesis I | 1,80E-01 |
| FOLSYN-PWY | superpathway of tetrahydrofolate biosynthesis and salvage | 9,75E-02 |
| GLCMANNANAUT-PWY | superpathway of N-acetylglucosamine, N-acetylmannosamine and N-acetylneuraminate degradation | 1,55E-01 |
| GLUCONEO-PWY | gluconeogenesis I | 9,73E-02 |
| GLYCOGENSYNTH-PWY | glycogen biosynthesis I (from ADP-D-Glucose) | 9,79E-02 |
| GLYCOLYSIS | glycolysis I (from glucose 6-phosphate) | 1,82E-01 |
| HISTSYN-PWY | L-histidine biosynthesis | 1,82E-01 |
| HOMOSER-METSYN-PWY | L-methionine biosynthesis I | 1,82E-01 |
| ILEUSYN-PWY | L-isoleucine biosynthesis I (from threonine) | 9,75E-02 |
| LACTOSECAT-PWY | lactose and galactose degradation I | 1,86E-01 |
| MET-SAM-PWY | superpathway of S-adenosyl-L-methionine biosynthesis | 1,31E-01 |
| NONOXIPENT-PWY | pentose phosphate pathway (non-oxidative branch) | 1,82E-01 |
| OANTIGEN-PWY | O-antigen building blocks biosynthesis (E. coli) | 9,82E-02 |
| P161-PWY | acetylene degradation | 1,88E-01 |
| P4-PWY | superpathway of L-lysine, L-threonine and L-methionine biosynthesis I | 1,57E-01 |
| P441-PWY | superpathway of N-acetylneuraminate degradation | 9,76E-02 |
| PEPTIDOGLYCANSYN-PWY | peptidoglycan biosynthesis I (meso-diaminopimelate containing) | 1,82E-01 |
| PHOSLIPSYN-PWY | superpathway of phospholipid biosynthesis I (bacteria) | 1,82E-01 |
| POLYISOPRENSYN-PWY | polyisoprenoid biosynthesis (E. coli) | 1,83E-01 |
| PROTOCATECHUATE-ORTHO-CLEAVAGE-PWY | protocatechuate degradation II (ortho-cleavage pathway) | 1,89E-01 |
| PWY-2942 | L-lysine biosynthesis III | 1,82E-01 |
| PWY-3001 | superpathway of L-isoleucine biosynthesis I | 9,75E-02 |
| PWY-5097 | L-lysine biosynthesis VI | 1,82E-01 |
| PWY-5100 | pyruvate fermentation to acetate and lactate II | 1,82E-01 |
| PWY-5101 | L-isoleucine biosynthesis II | 1,05E-01 |
| PWY-5103 | L-isoleucine biosynthesis III | 1,01E-01 |
| PWY-5104 | L-isoleucine biosynthesis IV | 9,75E-02 |
| PWY-5347 | superpathway of L-methionine biosynthesis (transsulfuration) | 9,90E-02 |
| PWY-5484 | glycolysis II (from fructose 6-phosphate) | 1,82E-01 |
| PWY-5667 | CDP-diacylglycerol biosynthesis I | 1,83E-01 |
| PWY-5686 | UMP biosynthesis | 1,83E-01 |
| PWY-5695 | urate biosynthesis/inosine 5'-phosphate degradation | 1,82E-01 |
| PWY-5973 | cis-vaccenate biosynthesis | 1,84E-01 |
| PWY-6121 | 5-aminoimidazole ribonucleotide biosynthesis I | 9,75E-02 |
| PWY-6122 | 5-aminoimidazole ribonucleotide biosynthesis II | 9,75E-02 |
| PWY-6123 | inosine-5'-phosphate biosynthesis I | 9,77E-02 |
| PWY-6125 | superpathway of guanosine nucleotides de novo biosynthesis II | 1,85E-01 |
| PWY-6126 | superpathway of adenosine nucleotides de novo biosynthesis II | 1,82E-01 |
| PWY-6147 | 6-hydroxymethyl-dihydropterin diphosphate biosynthesis I | 1,85E-01 |
| PWY-6151 | S-adenosyl-L-methionine cycle I | 1,82E-01 |
| PWY-6163 | chorismate biosynthesis from 3-dehydroquinate | 1,83E-01 |
| PWY-621 | sucrose degradation III (sucrose invertase) | 1,88E-01 |
| PWY-6277 | superpathway of 5-aminoimidazole ribonucleotide biosynthesis | 9,75E-02 |
| PWY-6383 | mono-trans, poly-cis decaprenyl phosphate biosynthesis | 1,55E-01 |
| PWY-6385 | peptidoglycan biosynthesis III (mycobacteria) | 1,82E-01 |
| PWY-6386 | UDP-N-acetylmuramoyl-pentapeptide biosynthesis II (lysine-containing) | 1,82E-01 |
| PWY-6387 | UDP-N-acetylmuramoyl-pentapeptide biosynthesis I (meso-diaminopimelate containing) | 1,82E-01 |
| PWY-6397 | mycolyl-arabinogalactan-peptidoglycan complex biosynthesis | 1,59E-01 |
| PWY-6609 | adenine and adenosine salvage III | 1,88E-01 |
| PWY-6612 | superpathway of tetrahydrofolate biosynthesis | 1,87E-01 |
| PWY-6737 | starch degradation V | 1,29E-01 |
| PWY-7007 | methyl ketone biosynthesis | 1,51E-01 |
| PWY-7111 | pyruvate fermentation to isobutanol (engineered) | 1,17E-01 |
| PWY-7196 | superpathway of pyrimidine ribonucleosides salvage | 1,01E-01 |
| PWY-7199 | pyrimidine deoxyribonucleosides salvage | 1,04E-01 |
| PWY-7200 | superpathway of pyrimidine deoxyribonucleoside salvage | 1,02E-01 |
| PWY-7208 | superpathway of pyrimidine nucleobases salvage | 1,83E-01 |
| PWY-7219 | adenosine ribonucleotides de novo biosynthesis | 1,82E-01 |
| PWY-7220 | adenosine deoxyribonucleotides de novo biosynthesis II | 1,82E-01 |
| PWY-7221 | guanosine ribonucleotides de novo biosynthesis | 1,82E-01 |
| PWY-7222 | guanosine deoxyribonucleotides de novo biosynthesis II | 1,82E-01 |
| PWY-7229 | superpathway of adenosine nucleotides de novo biosynthesis I | 1,82E-01 |
| PWY-7234 | inosine-5'-phosphate biosynthesis III | 1,83E-01 |
| PWY-7539 | 6-hydroxymethyl-dihydropterin diphosphate biosynthesis III (Chlamydia) | 1,82E-01 |
| PWY-7663 | gondoate biosynthesis (anaerobic) | 1,82E-01 |
| PWY-841 | superpathway of purine nucleotides de novo biosynthesis I | 9,75E-02 |
| PWY0-1061 | superpathway of L-alanine biosynthesis | 1,82E-01 |
| PWY0-1296 | purine ribonucleosides degradation | 1,82E-01 |
| PWY0-1298 | superpathway of pyrimidine deoxyribonucleosides degradation | 1,00E-01 |
| PWY0-1319 | CDP-diacylglycerol biosynthesis II | 1,83E-01 |
| PWY0-166 | superpathway of pyrimidine deoxyribonucleotides de novo biosynthesis (E. coli) | 1,85E-01 |
| PWY0-862 | (5Z)-dodec-5-enoate biosynthesis | 1,66E-01 |
| PWY1G-0 | mycothiol biosynthesis | 1,72E-01 |
| PWY4FS-7 | phosphatidylglycerol biosynthesis I (plastidic) | 1,83E-01 |
| PWY4FS-8 | phosphatidylglycerol biosynthesis II (non-plastidic) | 1,83E-01 |
| PWYG-321 | mycolate biosynthesis | 1,65E-01 |
| RIBOSYN2-PWY | flavin biosynthesis I (bacteria and plants) | 1,82E-01 |
| THRESYN-PWY | superpathway of L-threonine biosynthesis | 1,83E-01 |
| TRNA-CHARGING-PWY | tRNA charging | 1,88E-01 |
| TYRFUMCAT-PWY | L-tyrosine degradation I | 1,94E-01 |
| UDPNAGSYN-PWY | UDP-N-acetyl-D-glucosamine biosynthesis I | 1,82E-01 |
| VALSYN-PWY | L-valine biosynthesis | 9,75E-02 |
| ***MUC4* expression levels** | | |
| ARGORNPROST-PWY | arginine, ornithine and proline interconversion | 3,36E-02 |
| P4-PWY | superpathway of L-lysine, L-threonine and L-methionine biosynthesis I | 1,94E-02 |
| PPGPPMET-PWY | ppGpp biosynthesis | 1,38E-02 |
| PWY-1622 | formaldehyde assimilation I (serine pathway) | 4,12E-02 |
| PWY-5384 | sucrose degradation IV (sucrose phosphorylase) | 3,97E-02 |
| PWY-6628 | superpathway of L-phenylalanine biosynthesis | 2,05E-02 |
| PWY-6630 | superpathway of L-tyrosine biosynthesis | 3,65E-02 |
| ***MUC13* expression levels** | | |
| ARGSYN-PWY | L-arginine biosynthesis I (via L-ornithine) | 1,18E-02 |
| ARGSYNBSUB-PWY | L-arginine biosynthesis II (acetyl cycle) | 3,53E-02 |
| AST-PWY | L-arginine degradation II (AST pathway) | 1,30E-02 |
| CATECHOL-ORTHO-CLEAVAGE-PWY | catechol degradation to &beta;-ketoadipate | 4,85E-02 |
| COBALSYN-PWY | adenosylcobalamin salvage from cobinamide I | 6,48E-03 |
| GALACTARDEG-PWY | D-galactarate degradation I | 2,61E-02 |
| GLUCARDEG-PWY | D-glucarate degradation I | 3,53E-02 |
| GLUCARGALACTSUPER-PWY | superpathway of D-glucarate and D-galactarate degradation | 2,95E-02 |
| GLUTORN-PWY | L-ornithine biosynthesis | 3,95E-02 |
| GLYCOCAT-PWY | glycogen degradation I (bacterial) | 4,33E-02 |
| HISDEG-PWY | L-histidine degradation I | 2,42E-03 |
| P108-PWY | pyruvate fermentation to propanoate I | 3,54E-03 |
| P122-PWY | heterolactic fermentation | 6,47E-03 |
| P124-PWY | Bifidobacterium shunt | 4,59E-02 |
| P281-PWY | 3-phenylpropanoate degradation | 2,42E-02 |
| P4-PWY | superpathway of L-lysine, L-threonine and L-methionine biosynthesis I | 5,49E-03 |
| POLYAMSYN-PWY | superpathway of polyamine biosynthesis I | 1,38E-02 |
| PROTOCATECHUATE-ORTHO-CLEAVAGE-PWY | protocatechuate degradation II (ortho-cleavage pathway) | 1,30E-02 |
| PWY-181 | photorespiration | 3,14E-02 |
| PWY-5005 | biotin biosynthesis II | 2,49E-03 |
| PWY-5384 | sucrose degradation IV (sucrose phosphorylase) | 9,38E-03 |
| PWY-5417 | catechol degradation III (ortho-cleavage pathway) | 2,30E-02 |
| PWY-5431 | aromatic compounds degradation via &beta;-ketoadipate | 2,48E-02 |
| PWY-5505 | L-glutamate and L-glutamine biosynthesis | 2,18E-03 |
| PWY-5509 | adenosylcobalamin biosynthesis from cobyrinate a,c-diamide I | 4,20E-03 |
| PWY-5840 | superpathway of menaquinol-7 biosynthesis | 4,73E-02 |
| PWY-5910 | superpathway of geranylgeranyldiphosphate biosynthesis I (via mevalonate) | 3,84E-02 |
| PWY-6182 | superpathway of salicylate degradation | 2,82E-02 |
| PWY-6269 | adenosylcobalamin salvage from cobinamide II | 4,34E-03 |
| PWY-6317 | galactose degradation I (Leloir pathway) | 4,70E-02 |
| PWY-6470 | peptidoglycan biosynthesis V (&beta;-lactam resistance) | 3,87E-03 |
| PWY-6628 | superpathway of L-phenylalanine biosynthesis | 4,44E-03 |
| PWY-6630 | superpathway of L-tyrosine biosynthesis | 1,43E-03 |
| PWY-6876 | isopropanol biosynthesis | 2,67E-02 |
| PWY-6891 | thiazole biosynthesis II (Bacillus) | 2,16E-03 |
| PWY-6892 | thiazole biosynthesis I (E. coli) | 1,45E-03 |
| PWY-6895 | superpathway of thiamin diphosphate biosynthesis II | 1,12E-03 |
| PWY-7013 | L-1,2-propanediol degradation | 5,87E-04 |
| PWY-7328 | superpathway of UDP-glucose-derived O-antigen building blocks biosynthesis | 4,32E-02 |
| PWY-7376 | cob(II)yrinate a,c-diamide biosynthesis II (late cobalt incorporation) | 8,56E-03 |
| PWY-7377 | cob(II)yrinate a,c-diamide biosynthesis I (early cobalt insertion) | 8,69E-04 |
| PWY-7400 | L-arginine biosynthesis IV (archaebacteria) | 1,14E-02 |
| PWY0-1338 | polymyxin resistance | 2,90E-02 |
| PWY0-1533 | methylphosphonate degradation I | 2,90E-02 |
| PWY0-781 | aspartate superpathway | 8,24E-04 |
| PWY490-3 | nitrate reduction VI (assimilatory) | 2,35E-03 |
| PYRIDNUCSAL-PWY | NAD salvage pathway I | 1,77E-02 |
| PYRIDNUCSYN-PWY | NAD biosynthesis I (from aspartate) | 3,09E-02 |
| THISYN-PWY | superpathway of thiamin diphosphate biosynthesis I | 1,42E-03 |
| TYRFUMCAT-PWY | L-tyrosine degradation I | 2,31E-02 |
